# Hydroxamic Acid-Modified Peptide Library Provides Insights into the Molecular Basis for the Substrate Selectivity of HDAC Corepressor Complexes

**DOI:** 10.1021/acschembio.2c00510

**Published:** 2022-08-16

**Authors:** Lewis J. Archibald, Edward A. Brown, Christopher J. Millard, Peter J. Watson, Naomi S. Robertson, Siyu Wang, John W. R. Schwabe, Andrew G. Jamieson

**Affiliations:** † School of Chemistry, Advanced Research Centre, University of Glasgow, Glasgow G11 6EW, U.K.; ‡ The Leicester Institute of Structural and Chemical Biology, Department of Molecular and Cell Biology, University of Leicester, Leicester LE1 7RH, U.K.; § Department of Chemistry, University of Cambridge, Cambridge CB2 1GA, U.K.

## Abstract

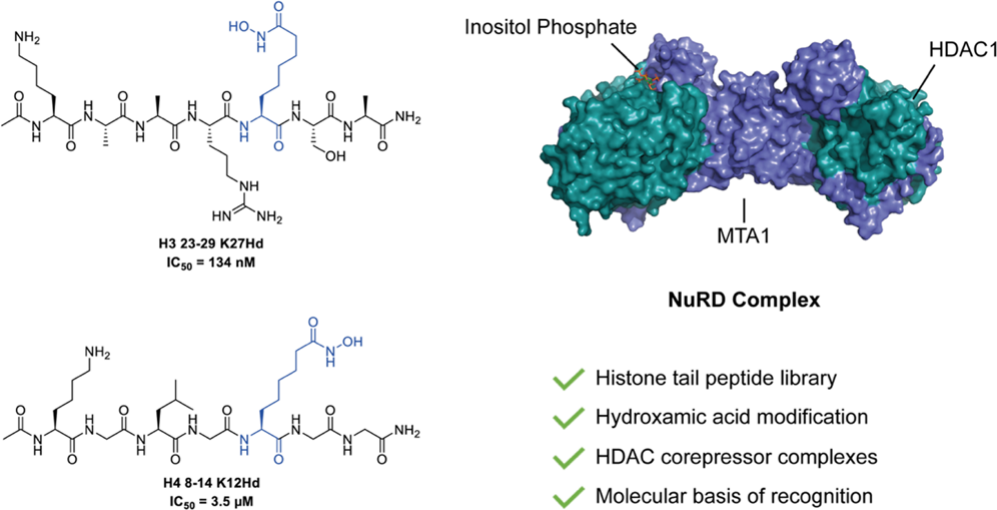

Targeting the lysine deacetylase activity of class I histone deacetylases (HDACs) is potentially beneficial for the treatment of several diseases including human immunodeficiency virus (HIV) infection, Alzheimer’s disease, and various cancers. It is therefore important to understand the function and mechanism of action of these enzymes. Class I HDACs act as catalytic components of seven large, multiprotein corepressor complexes. Different HDAC corepressor complexes have specific, nonredundant roles in the cell. It is likely that their specific functions are at least partly influenced by the substrate specificity of the complexes. To address this, we developed chemical tools to probe the specificity of HDAC complexes. We assessed a library of acetyl-lysine-containing substrate peptides and hydroxamic acid-containing inhibitor peptides against the full range of class I HDAC corepressor complexes. The results suggest that site-specific HDAC corepressor complex activity is driven in part by the recognition of the primary amino acid sequence surrounding a particular lysine position in the histone tail.

## Introduction

Class 1 histone deacetylases (HDACs) play an important role in the regulation of gene expression. They do so by removing acetyl modifications from lysine residues on the N-terminal tails of histones. Deacetylation reintroduces a positive charge to the lysine residue, increasing the strength of the interaction between the nucleosome and the negatively charged phosphate backbone of DNA. Thus, HDACs can control the recruitment of other chromatin regulators and influence chromatin structure, thereby determining which genes are transcriptionally active and which are repressed.

**Figure 1:**
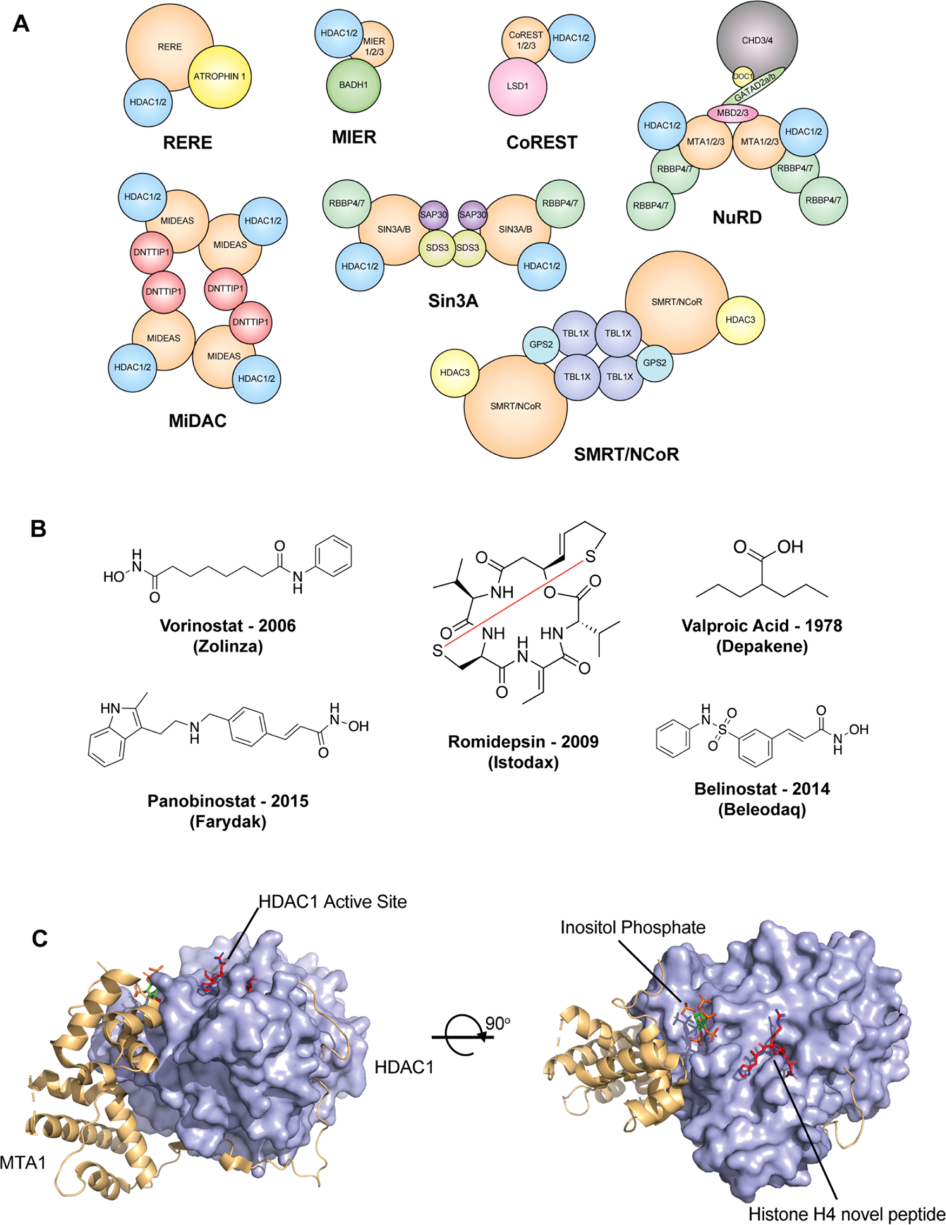
(A) Composition of the known class I HDAC corepressor complexes. (B) Chemical structures of U.S. Food and Drug Administration (FDA)-approved HDAC inhibitors. (C) X-ray crystal structure of an HDAC1/metastasis-associated protein 1 (MTA1) construct in complex with inositol phosphate and H4(12–18)K16Hd ligands (PDB:5ICN).

Class 1 HDACs 1–3 are recruited into large, multiprotein complexes that activate the enzyme and are thought to direct it toward its substrate. There are seven currently known complexes containing class-1 HDACs (Figure 1A). Arginine glutamic acid repeat (RERE), mesoderm induction early response (MIER), REST co-repressor (CoREST), nucleosome remodeling deacetylase (NuRD), and mitotic deacetylase complex (MiDAC) interact with HDAC1 and HDAC2 *via* their ELM2SANT domains; Sin3A is unique as it interacts with HDAC1 and HDAC2 through an HDAC interaction domain (HID) and lacks the SANT domain found in other complexes.^1−6^ The final complex, SMRT/NCoR, is the only complex that interacts with HDAC3 *via* the SANT-like deacetylase activation domain (DAD).^7^ The interaction between HDAC and the SANT domain in the corepressor protein forms a binding pocket for a higher order inositol phosphate, which increases the deacetylation activity of HDAC (Figure 1C).^7,8^

HDAC inhibitors have been used for the treatment of various forms of cancer, neurological disorders, and human immunodeficiency virus (HIV) infection.^9^ There are five HDAC inhibitors currently approved by the FDA, with a further 20 in various stages of clinical trials (Figure 1B).^10−12^ However, a fundamental issue with current HDAC inhibitor technologies is the lack of isoform or complex selectivity. This so-called “pan-HDAC” inhibition leads to undesired, off-target effects.^13^

In the known structures of HDAC corepressor complexes, the HDAC active site is oriented away from the interacting coregulator.^5,7,14,15^ Therefore, the immediate environment surrounding the HDAC active site is largely solvent accessible (Figure 1C).^5,15,16^ However, chemo-proteomic profiling of HDAC inhibitors has revealed selectivity against specific complexes. Bantscheff et al. assessed the complex selectivity of a range of known HDAC inhibitors by a combination of affinity capture and mass spectrometry.^17^ The authors found that inhibitors incorporating a benzamide zinc-binding group displayed low-micromolar affinity toward HDAC3–NCoR; however, no activity was found against the HDAC1/2-containing Sin3 complex. In addition, the bicyclic peptide romidepsin preferentially inhibited CoREST over NuRD and Sin3 despite sharing the same HDAC enzyme.^17^

A study by Wang et al. showed that HDAC complexes show sequence preference toward acetylated lysines in different positions in the nucleosome histone tails.^18^ MiDAC exhibited a 25-fold higher activity against H3K9ac over H3K23ac. CoREST displayed a similar deacetylation activity toward H3K9ac, H3K18ac, H3K23ac, and H3K27ac, with significantly lower activity against H3K14ac.

In 2016, we reported an H4(12–18)K16Hd peptide in which K16 was substituted for a hydroxamic acid (Hd) group. This peptide was found to be a nanomolar inhibitor of the HDAC1/MTA1 corepressor complex.^19^ An X-ray crystal structure of the peptide bound to this complex revealed several complementary interactions between the HDAC enzyme and the peptide backbone. While providing promising information regarding the influence of the histone tail peptide sequence on the substrate selectivity of the HDAC complex, the usefulness of the H4(12–18)K16Hd peptide was hindered by a lengthy, multistep synthesis.

Here, we describe the synthesis of a library of histone tail peptides based around known sites of lysine acetylation/deacetylation on H3 and H4, incorporating both acetyl-lysine and hydroxamic acid functionalities. We used this library to perform rate-of-turnover measurements, inhibition assays, and fluorescence polarization (FP) binding studies with HDAC complexes *in vitro* to elucidate the molecular basis of their substrate selectivity. We believe that this work sheds light on the effect of the primary amino acid sequence of the histone tail on the substrate selectivity of the HDAC complex, provides validation of hydroxamic acid functionality as an inhibitory mimic of acetyl-lysine, and reveals some of the key amino acid residues involved in the recognition of specific histone tail-lysine residues by HDAC corepressor complexes.

## Results and Discussion

### Fmoc/*^t^*Bu Solid-Phase Peptide Synthesis (Fmoc-SPPS) of the Histone Tail Peptide Library

To investigate whether the substrate selectivity of HDAC corepressor complexes is driven by the local amino acid sequence of histone N-terminal tails, we synthesized a library of short acetyl-lysine and hydroxamic acid-containing histone tail peptides. This library was tested *in vitro* against recombinantly expressed and purified HDAC complexes, primarily the HDAC1/MTA1(aa: 162–546)/RBBP4 core NuRD complex (abbreviated as HMR). By assessing the preference of this complex for some sequences over others, we hypothesized that we would be able to determine the molecular basis of recognition between the complex and the substrate/inhibitor peptide.

**Scheme 1:**
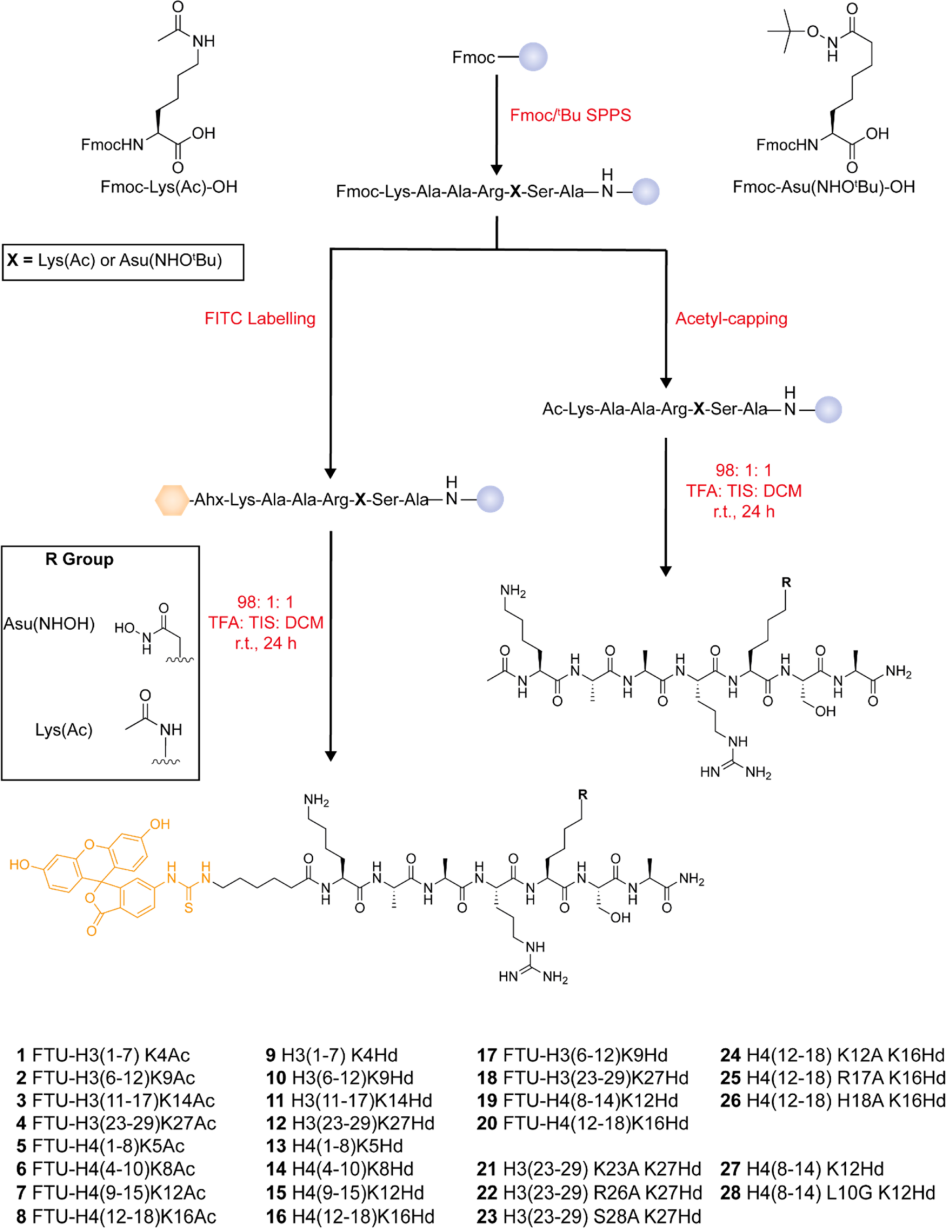
General Synthesis of Histone Tail Peptides Incorporating Acetyl-lysine or Asu(NHOH) Residues with and without N-Terminal Fluorescein Labels Chemical structures of Fmoc-Lys(Ac)-OH and Fmoc-Asu(NHO*^t^*Bu)-OH amino acid building blocks used are given on the top left and top right, respectively.

Peptides were synthesized by Fmoc/*^t^*Bu solid-phase peptide synthesis (Fmoc-SPPS) using Fmoc-Lys(Ac)-OH and an Fmoc-Asu(NHO*^t^*Bu)-OH building block, the synthesis of which we have previously reported (Scheme 1).^20^ A Rink amide resin was employed to leave the C-terminal amino functionality in each case, and non-fluorescein-labeled peptides were acetyl-capped at the N-terminus to replicate the lack of charge at either of these sites in the wider context of the entire histone sequence. In fluorescein-labeled peptides, an N-terminal 6-aminohexanoic acid (Ahx) linker was used to distance the fluorophore from the peptide sequence. Complete removal of the robust hydroxamic acid residue *tert*-butyl-protecting group was carried out in a trifluoroacetyl (TFA)/triisopropylsilane (TIS)/anhydrous dichloromethane (DCM) (98:1:1) cocktail for 24 h as part of the simultaneous cleavage of the peptide from the resin and global side-chain deprotection.

### Rate-of-Turnover of Substrate Peptides by HMR

**Figure 2:**
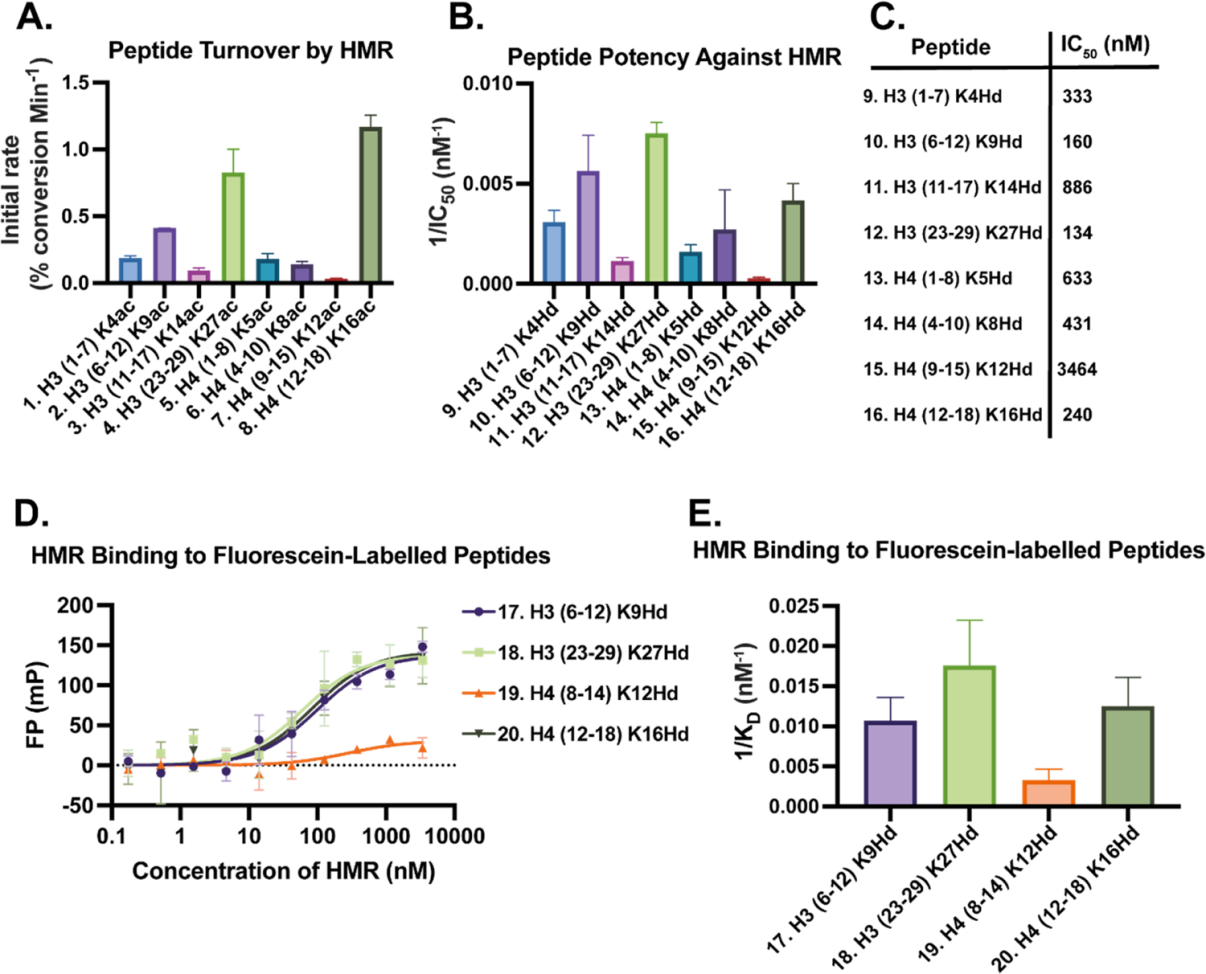
(A) Relative rate-of-turnover of the substrate peptide library by HMR. (B) Comparison of the inverse potencies (1/IC_50_) of the inhibitor peptide library against HMR. (C) Numerical IC_50_ values recorded for each of the inhibitor peptides. (D) FP binding data recorded for the fluorescein-labeled inhibitor peptides. (E) Comparison of the inverse binding constant (*K*_D_) determined for each of the fluorescein-labeled inhibitor peptides. Assays conducted with technical replicates *N* = 2.

The acetyl-lysine-containing library (compounds **1**–**8**) was assessed for the initial rate at which they were deacetylated by the HMR complex (Figure 2A).

The H4(12–18)K16Ac substrate peptide **8** was found to have the highest initial rate of deacetylation by the HMR complex, with the H3(23–29)K27Ac peptide **4** having the second highest rate. This result was expected given that the histone acetyl transferase (HAT)/HDAC activity at these histone lysine sites is known to be important in controlling chromatin architecture.^21−24^ The H3(6–12)K9Ac peptide **2** had the next highest initial rate of deacetylation. In contrast, H3(1–7)K4Ac **1** and H3(11–17)K14Ac **3** as well as H4(1–8)K5Ac **5** and H4(4–10)K8Ac **6** displayed moderate initial turnover rates. The H4(9–15)K12Ac peptide **7** was found to be the poorest substrate.

### Potency of Inhibitor Peptides toward the HMR Complex

An analogous library of hydroxamic acid-containing peptides spanning the same histone tail residues, with the hydroxamic acid-containing residue in the same position as acetyl-lysine in each case, was synthesized for comparison (compounds **9**–**16**) (Scheme 1). The hydroxamic acid functional group has been previously proven to be a useful tool for exploring the chemical biology of HDACs.^25−27^ These hydroxamic-acid-containing peptides were assessed for their potency of inhibition of the HMR complex (Figure 2B).

All of the inhibitor peptides, with the exception of H4(9–15)K12Hd (**15**), were found to inhibit the deacetylase activity of the HMR complex with nanomolar potency (Figure 2C). The H3(23–29)K27Hd **12** sequence was found to be the most potent among those tested. The relatively high potency observed for peptide **12** may correlate with the fact that H3K27Ac has been demonstrated to be a major substrate of the NuRD complex, of which HMR forms the core unit.^28^ Notably, H4(9–15)K12Hd **15** (the analogue of the poorest substrate peptide) was found to be the least potent inhibitor, with its IC_50_ value being approximately fourfold greater than that of the next least potent sequence, 3(11–17)K14Hd **11**.

Our primary observation from these data was a strong correlation between the substrate peptide turnover and inhibitor peptide potency, with a clear pattern observed across both assays. However, the H4(12–18)K16 sequence appeared to be a “better” substrate than an inhibitor, with the reverse being true for peptides based on H4(4–10)K8. This correlation is a significant finding that directly addresses the outstanding question of whether the substrate turnover of peptides of this type translates well into inhibitor potency, as posed by Moreno-Yruela et al. in their peptide microarray study.^29^ It also provides validation of the Asu(NHOH) side-chain as an effective substitute for acetyl-lysine for developing histone tail-mimetic peptide inhibitors. We therefore decided to focus our work on inhibitor peptides. We identified four key sequences for further study: H3(6–12)K9Hd **10**, H3(23–29)K27Hd **12**, H4(12–18)K16Hd **16**, and H4(9–15)K12Hd **15**. These sequences were chosen as they represent the three most potent and single least potent inhibitor peptides from the activity assays.

### Fluorescence Polarization Binding Studies

We aimed first to validate the results of the activity assay with the hydroxamic acid-containing peptides in terms of binding kinetics. To this end, we designed and synthesized fluorescein-labeled analogues (FTU) of the key peptides identified from the inhibition assay: FTU-H3(6−12)K9Hd **17**, FTU-H3(23–29)K27Hd **18**, FTU-H4(8−14)K12Hd **19**, and FTU-H4(12−18)K16Hd **20**. The H3(6–12)K9Hd and H4(9–15)K12Hd sequences from the original assay were revised to H3(6–12)K9Hd and H4(8–14)K12Hd respectively to match the other sequences with four residues N-terminal to the hydroxamic acid and two residues C-terminal to it. These peptides were then tested in an FP assay to measure their binding affinity for the HMR complex (Figure 2D).

All four of the labeled peptides displayed binding to the HMR complex. FTU-H3(23–29)K27Hd **18**, FTU-H4(12–18)K16Hd **20**, and FTU-H3(6–12)K9Hd **17** were observed to bind strongly to the HMR complex, correlating well with the high potency of their analogues (**12**, **16**, and **10**, respectively) in the activity assay. Interestingly, FTU-H3(6–12)K9Hd **17** was more potent than H4(12–18)K16Hd **16** in the inhibition assay, but the calculated *K*_D_ values for these two sequences in the FP assay were very similar. Unsurprisingly, the FTU-H4(8–14)K12Hd peptide **19** was found to be the “poorest” peptide among those tested (showing around threefold weaker binding compared with the other peptides) given the low potency of its corresponding analogue **15** in the activity assay.

These results validated the fact that the inhibitor potency observed in the activity assay indeed resulted from the binding of the peptide to the HDAC complex. This, in combination with the structure of the H4(12–18)K16Hd peptide in complex with HDAC1/MTA1, confirms that this class of peptides acts by blocking the HDAC catalytic site of the corepressor complex.^19^ In addition, the fact that the analogues of the three most potent sequences from the activity assay displayed strong binding (with the least potent peptide displaying much weaker binding in comparison) demonstrated that the addition of a linker and fluorophore to the N-terminus of the peptides did not significantly alter their ability to interact with the HMR complex.

### H3(23–29)K27Hd and H4(12–18)K16Hd Alanine Scan Experiments

With this validation in hand, we directed our attention toward investigating in more detail the effect of the primary amino acid sequence on the interaction with the HMR complex. For this, we decided to focus on the H3(23–29)K27Hd **12** and H4(12–18)K16Hd **16** peptides. The acetyl-lysine substrate analogues of these sequences were preferentially deacetylated in the catalytic turnover assay and, as previously stated, the H3K27 and H4K16 positions are known in the literature to be of relative importance in determining chromatin architecture.

**Figure 3:**
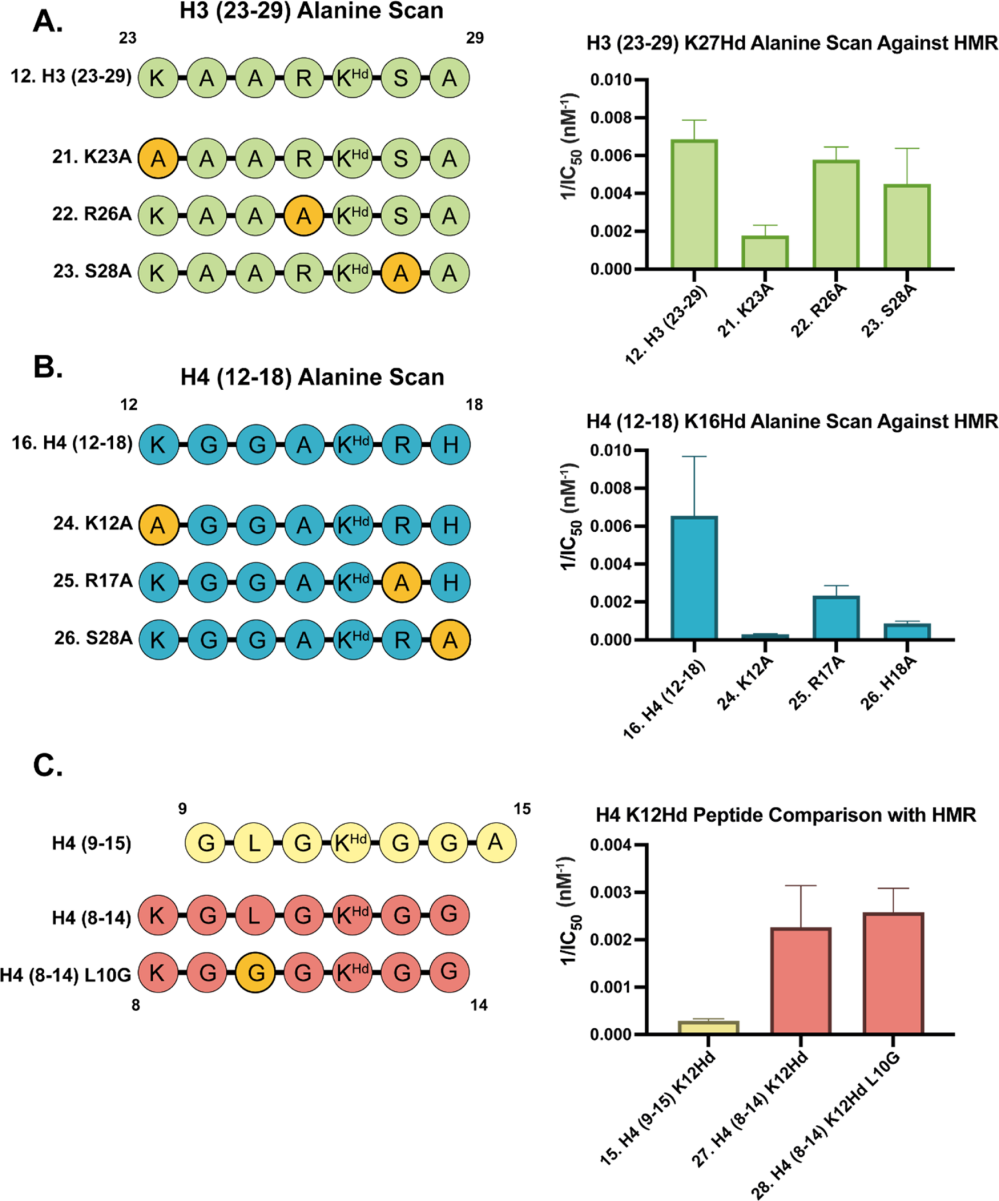
(A) Sequences of the H3(23–29)K27Hd alanine scan library (left) and a comparison of the inverse potency (1/IC_50_) against HMR of the compounds therein (right). (B) Sequences of the H4(12–18)K16Hd alanine scan library (left) and a comparison of the inverse potency (1/IC_50_) against HMR of the compounds therein (right). (C) Sequences of H4(9–15)K12Hd **15**, H4(8–14)K12Hd **27**, and H4(12–18)L10GK12Hd **28** (left) and a comparison of their inverse potency against HMR (right). Assays conducted with technical replicates *N* = 2.

We hypothesized that by performing an “alanine scan” of both the H3(23–29)K27Hd and H4(12–18)K16Hd sequences (in which alanine substitutions of the functional residues are made in a systematic fashion) and measuring their potency against HMR, we would elucidate the residues in each sequence that are key to their interaction with the complex. Three analogues of the H3(23–29)K27Hd sequence incorporating K23A, R26A, and S28A mutations (peptides **21**, **22**, and **23**) and three analogues of H4(12–18)K16Hd incorporating K12A, R17A, and H18A mutations (peptides **24**, **25**, and **26**) were synthesized, and their potencies against HMR were tested (Figure 3A,B, respectively).

The importance of proximal arginine residues in determining the selectivity of the HDAC complex for certain histone tail lysine sites was recently demonstrated by Wang et al. in their study on the catalytic activity of HDAC corepressor complexes on site-specifically acetylated nucleosomes.^18^ Our initial hypothesis therefore was that as both sequences contain an arginine residue directly adjacent to the hydroxamic acid, these residues would be the most important in maintaining the potency of the inhibitor.

Surprisingly, we observed the most significant decrease in the inhibitor potency for the H3(23–29)K27Hd sequence when lysine 23 was substituted for alanine (Figure 3A). The H3(23–29)K23A K27Hd peptide **21** was found to inhibit HMR with around fourfold less potency than the parent sequence. In comparison, the H3(23–29)R26A K27Hd analogue **22** displayed the highest potency among the three alanine scan analogues and was the closest to the parent sequence. This suggests that, in the context of histone tail peptides, a free lysine residue at position 23 may be of greater importance than the proximal arginine at position 26 in maintaining the potency of H3(23–29)K27Hd against HMR.

A strikingly similar pattern was observed in the potency of the alanine scan analogues of H4(12–18)K16Hd (compounds **24**–**26**) against the HMR complex (Figure 3B). Although all three analogues were less potent compared with the parent sequence, again, the most drastic decrease in potency was recorded for the sequence in which the N-terminal lysine residue was substituted with alanine. As with the H3(23–29)K27 sequence, in H4(12–18)K16Hd **16**, this lysine residue occupies the position *i*-4 relative to the hydroxamic acid. The results of these alanine scan experiments suggest a key role of the lysine residue in the *i*-4 position for directing the HMR complex activity to the H3K27 and H4K16 positions, respectively.

In addition to probing important residues in the H3(23–29)K27 and H4(12–18)K16 sequences, from which the “best” substrate peptides and two of the most potent inhibitor peptides were derived, we were also interested in exploring the relatively poor performance of peptides based on H4(8–15)K12. To address this issue, we synthesized and tested both H4(8–14)K12Hd **27** and H4(8–14)L10GK12Hd **28** against HMR to determine whether or not the low potency of **27** was driven by the steric repulsion caused by Leu10 (Figure 3C).

Both peptides **27** and **28** displayed very similar activities against HMR, with only a very subtle increase in potency observed for the L10G mutant relative to the parent sequence. However, when compared with the original H4(9–15)K12Hd **15** peptide (in which H4K8 was omitted), it was noted that both peptides based on H4(8–14)K12 had significantly higher potencies. This again implies an important role of the lysine residue *i*-4 to the hydroxamic acid in improving the interaction with HMR.

### Determination of the Selectivity of H3(23–29)K27Hd and H4(12–18)K16Hd to HDAC Complexes

**Figure 4:**
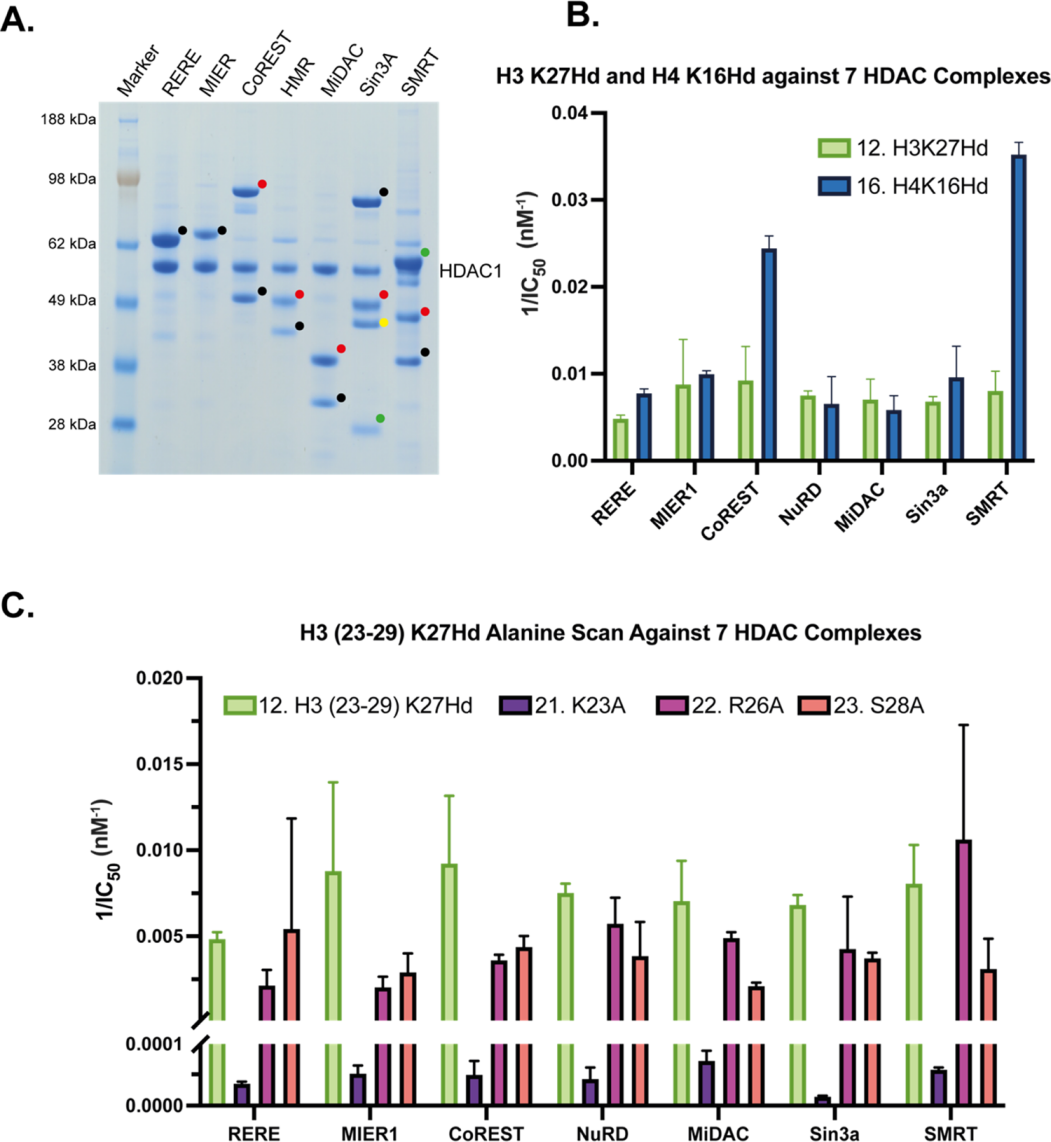
(A) Sodium dodecyl sulfate polyacrylamide gel electrophoresis (SDS-PAGE) analysis of each of the class I HDAC corepressor complexes used in these experiments: RERE (black dot = RERE), MIER1 (black dot = MIER1), CoREST (black dot = RCOR1, red dot = LSD1), HMR (black dot = MTA1, red dot = RBBP4), MiDAC (black dot = mitotic deacetylase-associated SANT domain (MIDEAS), red dot = DNTTIP1), Sin3A (black dot = Sin3A, red dot = RBBP4, green dot = SAP30L, yellow dot = SDS3), and SMRT (black dot = SMRT/GPS2 chimera, red dot = HDAC3, green dot = TBL1). (B) Inverse potencies (1/IC_50_) of H3(23–29)K27Hd **12** and H4(12–18)K16Hd **16** against all seven corepressor complexes. (C) Inverse potencies (1/IC_50_) of the alanine scan analogues of H3(23–29)K27Hd **12** against each of the corepressor complexes. Assays conducted with technical replicates *N* = 2.

Finally, we directed our attention toward assessing how the results from the experiments with HMR would be common to the other known class I HDAC corepressor complexes. RERE, MIER1, CoREST, HMR, MiDAC, Sin3A, and SMRT were expressed and purified, and the concentrations were normalized based on the concentration of HDAC1 or HDAC3 (Figure 4A). We determined the potency of H3(23–29)K27Hd **12** and H4(12–18)K16Hd **16** against RERE, MIER1, CoREST, NuRD, MiDAC, Sin3A, and SMRT (Figure 4B). In addition, we repeated the H3(23–29)K27Hd alanine scan experiment for each of these complexes to assess whether or not the importance of Lys23 for maintaining potency against HMR was replicated across the other complexes (Figure 4C).

No significant complex selectivity was observed for H3(23–29)K27Hd **12** (Figure 4B). Although the lowest potency of this peptide was recorded for the RERE complex, its activity against the remaining six corepressors was broadly similar. However, this was not the case for H4(12–18)K16Hd **16** (Figure 4B). Of the complexes tested, peptide **16** was found to inhibit SMRT with the highest potency. This is notable as SMRT is the only corepressor complex that contains HDAC3. The second-highest potency for **16** was recorded for the CoREST complex, which has inhibited ∼2.5-fold more strongly than MIER1, the next most potently inhibited complex. This difference is remarkable considering that CoREST shares the same interchangeable HDAC1/2 deacetylase component as MIER1. This implies that the other, non-HDAC components of the CoREST complex influence the HDAC catalytic site in such a way as to affect the binding of H4(12–18)K16Hd **16**.

For H3(23–29)K27Hd **12** and its alanine scan analogues (**21**–**23**), the significance of Lys23 in maintaining the potency of the peptide against HMR was also found to be true for the other corepressor complexes (Figure 4C). For each of the complexes tested, substitution of Lys23 with alanine resulted in a drastic decrease in activity relative to the parent sequence. This again demonstrates the importance of this residue particularly in driving the binding of this sequence to an HDAC corepressor complex.

## Conclusions

In conclusion, we demonstrated how a small library of substrate and inhibitor peptides derived from histone tails can provide insights into how these sequences are recognized by HDAC corepressor complexes. We showed that the rate-of-turnover of acetyl-lysine-containing substrate peptides correlates well with the potency of analogous inhibitor peptides, addressing an outstanding question in the field. We validated these results in terms of binding affinity using a fluorescence-polarization assay of N-terminally labeled analogues.

In addition, we identified the importance of the lysine residue *i*-4 to the hydroxamic acid in determining the potency of H3(23–29)K27Hd **12**, H4(9–15)K12Hd **15**, and H4(12–18)K16Hd **16**. We also explored how the significance of Lys23 with respect to the potency of **12** applies to each of the known class I HDAC corepressor complexes. Finally, we showed that the H4(12–18)K16Hd **16** peptide is capable of inhibiting the CoREST and SMRT complexes more strongly than the remaining five corepressors, suggesting that complex-selective inhibition is possible with peptides of this size as well as implying a preference of these HDAC corepressor complexes for this lysine position. In conclusion, the data presented provide strong evidence that site-specific activity of HDAC corepressor complexes is driven, in part, by the recognition of the primary amino acid sequence surrounding a particular histone tail lysine site.

## Methods

### General Information

All amino acids are of L-configuration unless otherwise stated. Standard Fmoc-protected amino acids were purchased from CEM Corporation or Pepceuticals. Peptide-grade dimethylformamide (DMF) was purchased from Rathburn. Peptides were synthesized on a Biotage Initiator+ Alstra microwave-assisted peptide synthesizer. Peptides were purified on a reverse-phase Dionex HPLC system equipped with Dionex P680 pumps and a Dionex UVD170U UV–vis detector (monitoring at 214 and 280 nm), using a Phenomenex, Gemini, C18, 5 μm, 250 × 21.2 mm^2^ column, a Phenomenex, Kinetex, C18, 5 μm, 250 × 10.0 mm^2^ column, or a ReproSil, Gold 200, C4, 5 μm, 250 × 20 mm^2^ column. Gradients were obtained using solvents consisting of A (H_2_O + 0.1% TFA) and B (MeCN + 0.1% TFA), and fractions were lyophilized on a Christ Alpha 2–4 LO plus freeze dryer. Pure peptides were analyzed on a Shimadzu reverse-phase HPLC (RP-HPLC) system equipped with Shimadzu LC-20AT pumps, a SIL-20A autosampler, and an SPD-20A UV–vis detector (monitoring at 214 and 280 nm) using a Phenomenex, Aeris, 5 μm, peptide XB-C18, 150 × 4.6 mm^2^ column at a flow rate of 1 mL/min or a ReproSil, Gold 200, C4, 5 μm, 250 × 4.6 mm^2^ column at a flow rate of 1 mL/min. RP-HPLC gradients were run using a solvent system consisting of solutions A (5% MeCN in H_2_O + 0.1% TFA) and B (5% H_2_O in MeCN + 0.1% TFA). Two gradients were used to characterize each peptide: a gradient from 0 to 100% solution B over 20 min and a gradient from 0–100% solution B over 50 min. Peptides **1**–**8** were characterized over analogous 15 and 30 min gradients. Analytical RP-HPLC data are reported as the column retention time (tR) in minutes (min). High-resolution mass spectrometry (HRMS) of pure peptides was performed on a Bruker microTOF-Q II (ESI+).

### Peptide Synthesis

#### Procedure for Automated Peptide Synthesis (Biotage Initiator+ Alstra Synthesizer)

Fmoc-protected amino acids were prepared as a 0.2 M solution in DMF. Amino acids (4 equiv relative to the resin loading) were used during coupling cycles, with the exception of Fmoc-Asu(NHO*^t^*Bu)-OH for which 2 equiv were used. HCTU was prepared as a 0.5 M solution in DMF, and *N*,*N*-diisopropylethylamine (DIPEA) was prepared as a 2 M solution in *N*-methyl-2-pyrrolidone (NMP). HCTU (4 equiv) and 8 equiv of DIPEA (relative to resin loading) were used during coupling cycles. For Fmoc deprotections, a solution of 20% piperidine in DMF was used. Coupling reactions were performed under microwave heating at 75 °C for 5 min with the exception of Fmoc-His(Trt)-OH, Fmoc-Arg(PBf)-OH, Fmoc-Lys(Ac)-OH, and Fmoc-Asu(NHO*^t^*Bu)-OH. Coupling of Fmoc-His(Trt)-OH was performed for 5 min at room temperature (rt) followed by 5 min at 50 °C. Coupling of Fmoc-Arg(Pbf)-OH was performed for 45 min at rt followed by 5 min at 75 °C. Coupling of Fmoc-Lys(Ac)-OH and Fmoc-AsuNHOH(*^t^*Bu)-OH was performed under microwave heating at 75 °C for 10 min. Standard Fmoc deprotections were carried out at rt for 3 and 10 min consecutively. Microwave-assisted Fmoc deprotections were carried out at 75 °C for 30 s, followed by a second deprotection at 75 °C for 3 min. For acetyl capping, acetic anhydride was made up to 5 M in DMF, and a solution of 2 M DIPEA in NMP was used as the base. Capping steps were performed at 75 °C for 10 min.

Typically, cleavage tests of peptides were performed by taking ∼3 mg of dried resin beads and treating them with TFA/TIS/water (95:2.5:2.5) for 2 h. Cleavage tests of peptides containing Asu(NHO*^t^*Bu) were performed by taking ∼3 mg of dried resin beads and treating them with TFA/TIS/DCM (98:1:1) for 24 h. The filtrate was drained, concentrated, and then triturated in cold diethyl ether (Et_2_O). The triturate was dissolved in acetonitrile/water and then analyzed by RP-HPLC/LC-MS.

### General Procedure for Manual Peptide Synthesis

Peptides were synthesized on a 0.1 mmol scale in a 20 mL fritted syringe using Fmoc-Rink Amide AM resin purchased from Iris Biotech (substitution: 0.74 mmol/g). Fmoc deprotection was carried out twice with a solution of 20% v/v piperidine in DMF (2 × 3.00 mL) with gentle rocking for 3 min and then 10 min, followed by sequential washing of the resin with DMF (3 × 3.00 mL) and DCM (3 × 3.00 mL).

Amino acid couplings were carried out using Fmoc-protected amino acid (4.00 equiv for natural or 2.00 equiv for unnatural relative to resin loading) and HCTU (4.00 equiv for natural or 2.00 equiv for unnatural relative to resin loading) dissolved in the minimum amount NMP and DIPEA (8.00 equiv for natural or 4.00 equiv for unnatural relative to resin loading). The resulting solution was allowed to activate for 5 min before addition to the prepared resin. The resin suspension was gently rocked for 2 h, and then the resin was drained and washed sequentially with DMF (3 × 3.00 mL) and DCM (3 × 3.00 mL). N-terminal acetyl capping was achieved using a mixture of DIPEA (50.0 equiv relative to resin loading) and acetic anhydride (Ac_2_O 50.0 equiv relative to resin loading) in DMF at ambient temperature for 10 min.

### General Procedure for TFA Cleavage of Peptides

Peptides were typically cleaved from the resin by gently rocking the resin at rt in a cleavage cocktail of TFA/TIS/H_2_O (95:2.5:2.5) for 2 h before being drained, and TFA was blown off with a steady stream of N_2_ gas. Peptides containing Asu(NHO*^t^*Bu) were cleaved from the resin using a cleavage cocktail of TFA/TIS/anhydrous DCM (98:1:1) for 24 h. In all cases, the crude peptide was triturated with cold Et_2_O. Et_2_O was removed from the resulting crude peptide pellet under a steady stream of nitrogen. The crude peptide was then redissolved in H_2_O/MeCN and purified by RP-HPLC.

### General Procedure for N-Terminal FITC-Labeling

N-terminal Fmoc-protected on-resin peptide was placed into a fritted syringe. The resin was allowed to swell in DCM for 20 min and then drained. Fmoc deprotection was achieved by sequential 3 and 10 min treatments with 20% piperidine in DMF followed by washing with DMF (3 × 3.00 mL) and then DCM (3 × 3.00 mL). Fluorescein isothiocyanate (isomer I, 2.00 equiv relative to resin substitution) and DIPEA (4.00 equiv relative to resin substitution) were dissolved in DMF; the mixture was added to the resin and gently rocked at rt for 3 h. Upon completion, the reaction vessel was drained, and the resin was washed with DMF (3 × 3.00 mL) and then DCM (3 × 3.00 mL).

#### Protein Expression and Purification

Each HDAC complex was expressed in a HEK293F cell expression system. For each 300 mL of cells (density of 1 × 10^6^ cells/mL) (1.2 L was prepared for each complex), a total of 300 μg of DNA was mixed with 600 μg of poly(ethylamine) (PEI) (Sigma) in 30 mL of phosphate-buffered saline (PBS) (Sigma). This transfection reaction mixture was vortexed and incubated for 20 min before being added to the cells. The cells were incubated for 48 h before harvesting and lysed by sonication in a buffer containing 50 mM Tris/HCl, pH 7.5, 150 mM KAc, 10% v/v glycerol, 0.3% v/v Triton X-100, and a complete EDTA-free protease inhibitor cocktail (Roche) (buffer A). The insoluble fraction was removed by centrifugation. The soluble fraction was then added to anti-Flag Agarose resin (Sigma) and incubated for 30 min at 4 °C. The complex was then washed three times with buffer A, three times with buffer B (50 mM Tris/HCl, pH 7.5, 150 mM KAc, 5% v/v glycerol), and five times with buffer C (50 mM Tris/HCl, pH 7.5, 50 mM KAc, 5% v/v glycerol, and 0.5 mM tris(2-carboxyethyl) phosphine–HCl (TCEP)). Tobacco etch virus (TEV) protease was added to release the complex from the resin.

The supernatant after TEV cleavage was concentrated and filtered before being loaded onto a size exclusion chromatography column (Superdex 200 10/300 (Cytiva) column for HMR, RERE, MIER1, MiDAC, and SMRT; Superose 6 10/300 (Cytiva) for CoREST and Sin3A (25 mM Tris/HCl, pH 7.5, 50 mM KAc, and 0.5 mM TCEP)), and the complex fractions were selected and concentrated for further experiments. The protein complexes were stored by flash freezing in liquid nitrogen in the presence of 25% glycerol before being transferred to a freezer at −80 °C.

#### Caliper Deacetylation Assays

Reactions (30 μL) contained 125 nM HMR and 2 μM fluorescein-labeled peptides (peptides 1–8) in 50 mM Tris/HCl, pH 7.5, 50 mM NaCl, and 5% glycerol. The deacetylase reaction was recorded over a 30 min period, every 90 s, using a Caliper EZ Reader II System (Caliper Life Sciences, http://www.caliperls.com). The initial rates were calculated using the formula *Y* = *Y*-intercept + slope × *X* during the first 8 min of the reaction using GraphPad Prism 9.

#### Fluorescence Polarization Assays

The fluorescence polarization assay was performed using 96-well black plates (Corning). FTU (10 nM)-labeled peptides (peptides 17–20) were incubated with increasing concentrations of HMR for 30 min at rt. The plate was shaken before being read on a Victor X5 Plate reader (Perkin Elmer). 1/*K*_D_ values were calculated using the nonlinear regression one-site binding equation *Y* = *B*_max_ × *X*/(*K*_D_ + *X*) using Graphpad Prism 9.

#### Boc-Lys HDAC Inhibition Assays

Inhibition assays with various peptide inhibitors were performed using a fluorescence-based assay. The inhibitor peptides were initially dissolved in 5% dimethyl sulfoxide (DMSO) at a stock concentration of 25 mM before being further diluted in the HDAC assay buffer (50 mM Tris/HCl, pH 7.5, 150 mM NaCl). Serial dilutions (1:3) of the inhibitor were prepared, starting at a concentration of 500 μM. HDAC complexes were diluted to a final concentration of 50 nM and incubated with the inhibitor for 20 min at rt. The Boc-(Ac)Lys-AMC substrate was added at a final concentration of 100 μM The final volume of the reaction was 50 μL. The reaction was incubated at 37 °C, 150 rpm, for 30 min, before a developer (50 mM Tris, pH 7.5, 100 mM NaCl, 10 mg/mL trypsin) was added. The reaction was incubated with the developer for 10 min before being measured (PerkinElmer, 2030 multilabel reader, VICTOR X5, excitation 335 nm, emission 460 nm). The absorbance of the buffer as the blank control was subtracted from the HDAC activity, and IC_50_ calculations were performed using the nonlinear regression log(inhibitor) vs response equation Y = bottom + (top – bottom)/(1+10^(*X*–Log IC_50_)^) in Graphpad Prism 9.

## Supporting Information Available

Peptide characterization data; HDAC corepressor complex components; primary assay data; and dose–response curves (PDF)

## Abbreviations

CoREST: REST co-repressor
DAD: deacetylase activation domain
DCM: dichloromethane
DNA: deoxyribonucleic acid
ELM2: Egl-27 and MTA1 homology 2
FDA: U.S. Food and Drug Administration
FP: fluorescence polarization
FTU: fluorescein thiourea
HAT: histone acetyl transferase
HDAC: histone deacetylase
HID: HDAC interaction domain
HIV: human immunodeficiency virus
MIDEAS: mitotic deacetylase-associated SANT domain
MIER1: mesoderm induction early response protein 1
MTA1: metastasis-associated protein 1
NCOR2: nuclear receptor corepressor 2 (SMRT)
NuRD: nucleosome remodeling deacetylase
RERE: arginine glutamic acid repeat encoding
TEV: tobacco etch virus
TFA: trifluoroacetic acid
TIS: triisopropyl silane
