# Supplementary material for: Hydroxamic Acid-Modified Peptide Library Provides Insights into the Molecular Basis for the Substrate Selectivity of HDAC Corepressor Complexes
Source: ACS Chem Biol. 2022 Aug 16;17(9):2572–82. doi: 10.1021/acschembio.2c00510 (PMC9488896; doi:10.1021/acschembio.2c00510)
Supplement: Supplementary file 1 — cb2c00510_si_001.pdf [file cb2c00510_si_001.pdf]

A Hydroxamic Acid-Modified Peptide Library Provides Insight into the Molecular Basis for Substrate Selectivity of HDAC Corepressor Complexes

Lewis J. Archibald^†, Edward A. Brown^‡, Christopher J. Millard‡, Peter J. Watson‡, Naomi S. Robertson₴, Siyu Wang‡, John W.R. Schwabe*‡ and Andrew G. Jamieson*†

† School of Chemistry, Advanced Research Centre, University of Glasgow, Glasgow, G11 6EW, U.K.

‡ The Leicester Institute of Structural and Chemical Biology, Department of Molecular and Cell Biology, University of Leicester, Leicester, LE1 7RH, U.K.

₴ Department of Chemistry, University of Cambridge, Cambridge, CB2 1GA, U.K.

Corresponding authors email:

john.schwabe@le.ac.uk

andrew.jamieson.2@glasgow.ac.uk

Supplementary Information

S.1 Peptide Characterisation Table

Sequence	Calculated $[M+nH]^{n+}$ (Da)	Found $[M+nH]^{n+}$ (Da)	Δ $[M+nH]^{n+}$ (ppm)	^{t}R (min)	Yield (%)	Purity (%)
FTU-H3(1-7)K4Ac<br>FTU-Ahx-ART**KAc**QTA-NH_2<br>**1**	$[M+H]^+$<br>1318.5890	$[M+H]^+$<br>1318.5922	2.4	15.2[a]<br>19.0[b]	30	99
FTU-H3(6-12)K9Ac<br>FTU-Ahx-TAR**KAc**STG-NH_2<br>**2**	$[M+H]^+$<br>1263.5468	$[M+H]^+$<br>1263.5522	4.3	14.5[a]<br>18.3[b]	27	98
FTU-H3(11-17)K14Ac<br>FTU-Ahx-TGG**KAc**APR-NH_2<br>**3**	$[M+H]^+$<br>1229.5414	$[M+H]^+$<br>1229.5468	4.4	14.6[a]<br>18.5[b]	28	98
FTU-H3(23-29)K27Ac	$[M+H]^+$	$[M+H]^+$	2.4	14.0[a]	45	99

FTU-Ahx-KAAR**KAc**SA-NH_2<br>**4**	1274.5992	1274.6022		17.6[b]		
FTU-H4(1-8)K5Ac<br>FTU-Ahx-SGRG**KAc**GGK-NH_2<br>**5**	$[M+H]^+$<br>1289.5737	$[M+H]^+$<br>1289.5824	6.7	14.0[a]<br>17.3[b]	26	91
FTU-H4(4-10)K8Ac<br>FTU-Ahx-GKGG**KAc**GL-NH_2<br>**6**	$[M+H]^+$<br>1159.5246	$[M+H]^+$<br>1159.5282	3.1	14.9[a]<br>19.0[b]	25	99
FTU-H4(9-15)K12Ac<br>FTU-Ahx-GLG**KAc**GGA-NH_2<br>**7**	$[M+H]^+$<br>1102.4668	$[M+H]^+$<br>1102.4729	5.5	15.0[a]<br>19.4[b]	41	99
FTU-Ahx-H4(12-18)K16Ac<br>FTU-Ahx-KGGA**KAc**RH-NH_2<br>**8**	$[M+H]^+$<br>1295.5864	$[M+H]^+$<br>1295.5897	2.5	13.8[a]<br>18.4[b]	31	99
H3(1-7)K4Hd<br>Ac-ART**Hd**QTA-NH_2<br>**9**	$[M+H]^+$<br>874.4741	$[M+H]^+$<br>874.4703	4.3	8.3[c]<br>11.9[d]	5	90
H3(6-12)K9Hd<br>Ac-TAR**Hd**STG-NH_2<br>**10**	$[M+H]^+$<br>819.4319	$[M+H]^+$<br>819.4292	3.3	8.0[c]<br>7.2[d]	16	96
H3(11-17)K14Hd<br>Ac-TGG**Hd**APR-NH_2<br>**11**	$[M+H]^+$<br>785.4264	$[M+H]^+$<br>785.4240	3.1	8.4[c]<br>12.3[d]	17	92
H3(23-29)K27Hd<br>Ac-KAAR**Hd**SA-NH_2	$[M+2H]^{2+}$<br>415.7458	$[M+2H]^{2+}$<br>415.7473	-3.6	8.3[c]<br>11.9[d]	11	97

**12**						
H4(1-8)K5Hd<br>Ac-SGRG**Hd**GGK-NH_2<br>**13**	$[M+H]^+$<br>845.4588	$[M+H]^+$<br>845.4599	-1.3	4.4[c]<br>5.0[d]	12	96
H4(4-10)K8Hd<br>Ac-GKGG**Hd**GL-NH_2<br>**14**	$[M+H]^+$<br>715.4097	$[M+H]^+$<br>715.4088	1.3	9.2[c]<br>14.1[d]	4	88
H4(9-15)K12Hd<br>Ac-GLG**Hd**GGA-NH_2<br>**15**	$[M+H]^+$<br>658.3519	$[M+H]^+$<br>658.3512	1.0	9.3[c]<br>14.4[d]	3	91
H4(12-18)K16Hd<br>Ac-KGGA**Hd**RH-NH_2<br>**16**	$[M+2H]^{2+}$<br>426.7436	$[M+2H]^{2+}$<br>426.7442	-1.5	7.4[c]<br>7.4[d]	25	94
FTU-H3(5-11)K9Hd<br>FTU-Ahx-QTAR**Hd**ST-NH_2<br>**17**	$[M+H]^+$<br>1350.5783	$[M+H]^+$<br>1350.5753	2.2	12.4[c]<br>22.2[d]	6	97
FTU-H3(23-29)K27Hd<br>FTU-Ahx-KAAR**Hd**SA-NH_2<br>**18**	$[M+2H]^{2+}$<br>645.8004	$[M+2H]^{2+}$<br>645.7981	3.7	12.1[c]<br>21.3[d]	11	98
FITC-H4(8-14)K12Hd<br>FTU-Ahx-KGLG**Hd**GG-NH_2<br>**19**	$[M+2H]^{2+}$<br>588.2631	$[M+2H]^{2+}$<br>588.2611	3.5	12.8[c]<br>23.3[d]	5	95
FTU-H4(12-18)K16Hd<br>FTU-Ahx-KGGA**Hd**RH-NH_2<br>**20**	$[M+2H]^{2+}$<br>656.7982	$[M+2H]^{2+}$<br>656.7966	2.5	11.8[c]<br>20.7[d]	3	>99

H4(8-14)K12Hd<br>Ac-KGLG**Hd**GG-NH_2<br>**21**	$[M+H]^+$<br>715.4097	$[M+H]^+$<br>715.4088	1.2	16.5[c]<br>30.1[d]	19	98
H4(8-14)L10GK12Hd<br>Ac-KGGG**Hd**GG-NH_2<br>**22**	$[M+H]^+$<br>659.3471	$[M+H]^+$<br>659.3493	-3.3	8.5[c]<br>12.8[d]	12	93
H3(23-29)K23AK27Hd<br>Ac-AAAR**Hd**SA-NH_2<br>**23**	$[M+H]^+$<br>773.4264	$[M+H]^+$<br>773.4255	1.2	8.2[c]<br>11.6[d]	8	94
H3(23-29)R26AK27Hd<br>Ac-KAAA**Hd**SA-NH_2<br>**24**	$[M+H]^+$<br>745.4203	$[M+H]^+$<br>745.4214	-1.5	8.1[c]<br>10.2[d]	16	94
H3(23-29)K27HdS28A<br>Ac-KAAR**Hd**AA-NH_2<br>**25**	$[M+H]^+$<br>814.4894	$[M+H]^+$<br>814.4904	-1.3	8.4[c]<br>12.4[d]	10	93
H4(12-18)K12AK16Hd<br>Ac-AGGA**Hd**RH-NH_2<br>**26**	$[M+H]^+$<br>795.4220	$[M+H]^+$<br>795.4246	-3.3	8.1[c]<br>11.1[d]	12	98
H4(12-18)K16HdR17A<br>Ac-KGGA**Hd**AH-NH_2<br>**27**	$[M+H]^+$<br>767.4159	$[M+H]^+$<br>767.4167	-1.1	8.8[c]<br>13.8[d]	11	91
H4(12-18)K16HdH18A<br>Ac-KGGA**Hd**RA-NH_2<br>**28**	$[M+H]^+$<br>786.4581	$[M+H]^+$<br>786.4604	-3.0	5.5[c]<br>5.6[d]	8	98

Analytical reverse-phase HPLC using a Shimadzu LC system with a Phenomenex Aeris™ 5 µm Peptide XB-C18 100Å packed column with dimensions of 4.6 x 150 mm, using the following gradients: [a]5-95% MeCN/H_2O (0.1% TFA), 15 min gradient, [b]5-95% MeCN/H_2O (0.1% TFA), 30 min gradient [c]5-95% MeCN/H_2O (0.1% TFA), 20 min gradient, [d]5-95% MeCN/H_2O (0.1% TFA), 50 min gradient.

S.2 Peptide Analytical RP-HPLC Traces

1 FTU-H3(1-7)K4Ac

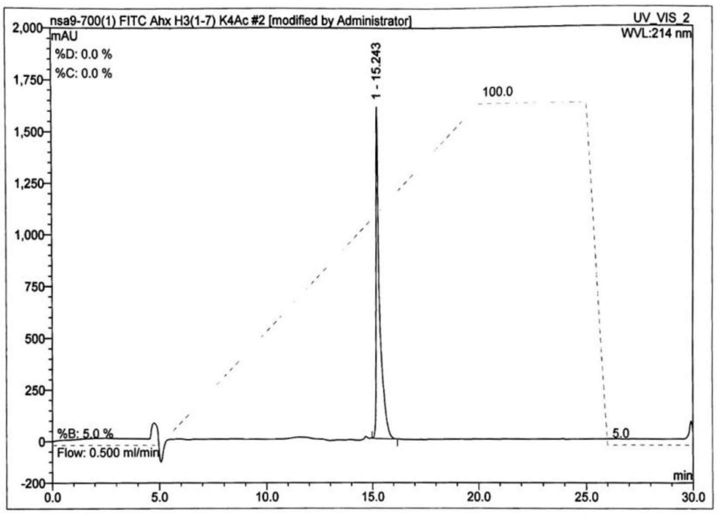

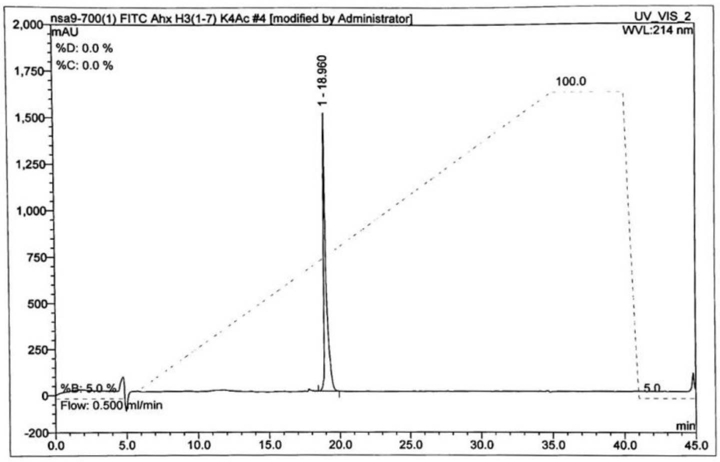

2 FTU-H3(6-12)K9Ac

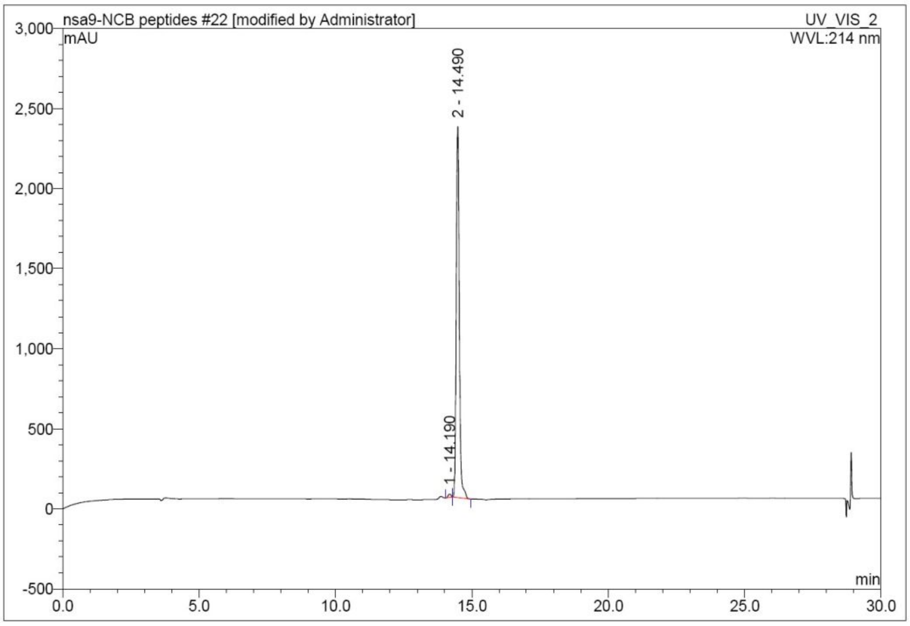

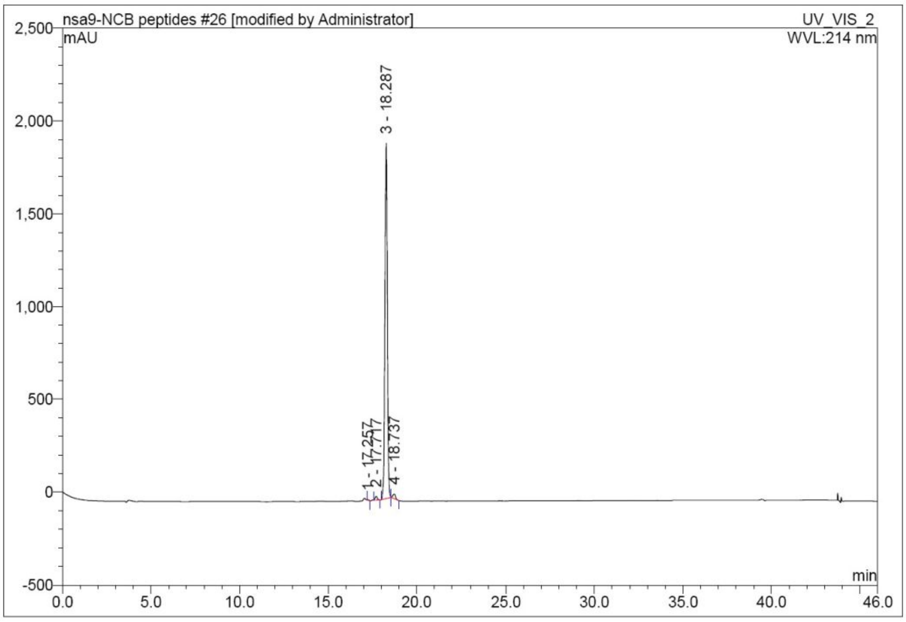

3 FTU-H3(11-17)K14Ac

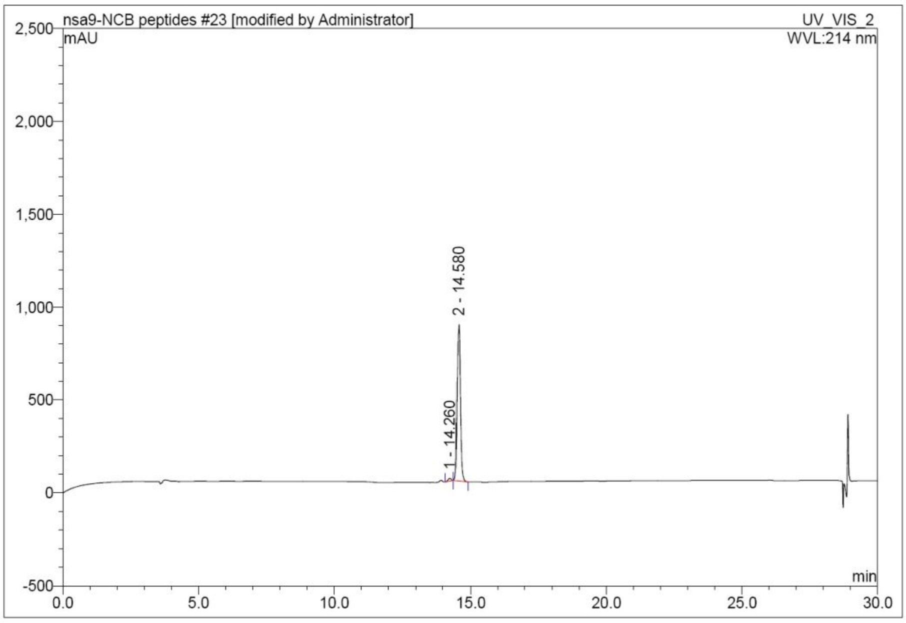

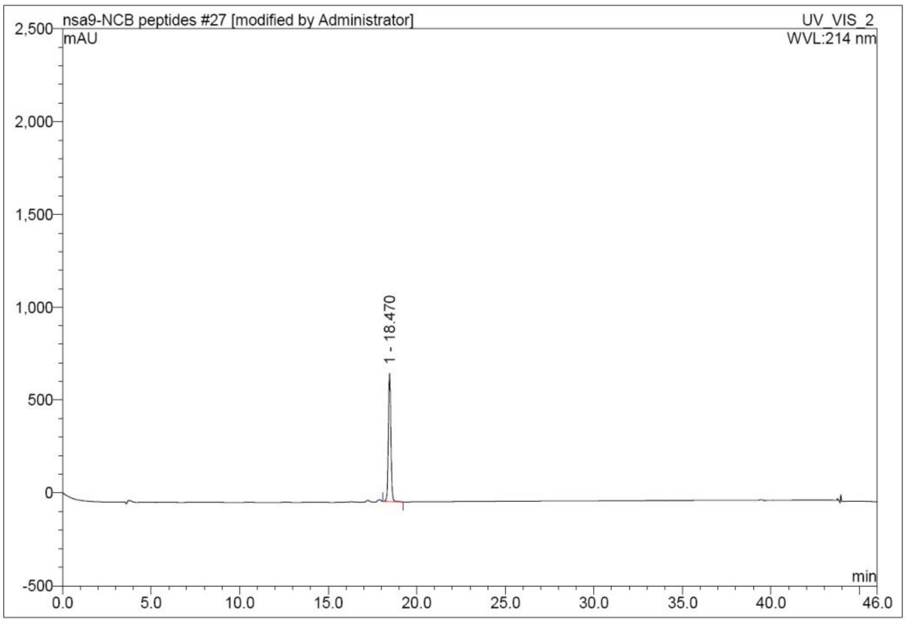

4 FTU-H3(23-29)K27Ac

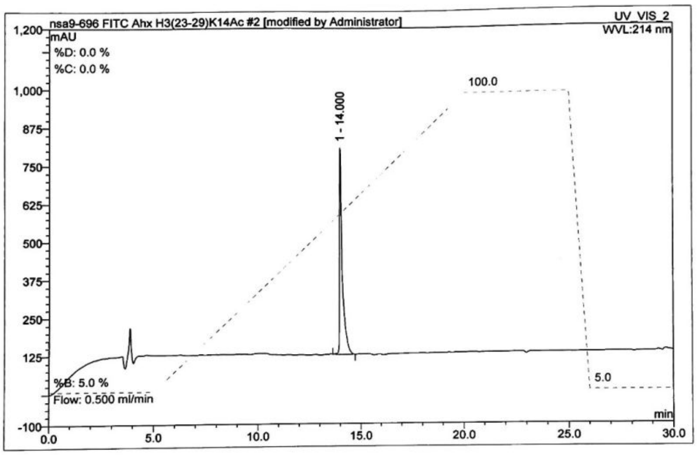

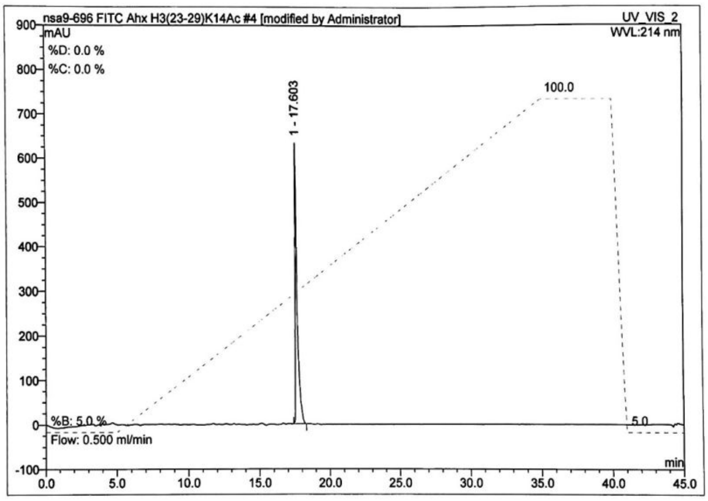

nsa9-696 FITC Ahx H3(23-29)K14Ac #4 [modified by Administrator]
UV_VIS_2
WVL:214 nm
mAU
%D: 0.0 %
%C: 0.0 %
1 - 17.603
100.0
%B: 5.0 %
Flow: 0.500 ml/min
5.0
min

5 FTU-H4(1-8)K5Ac

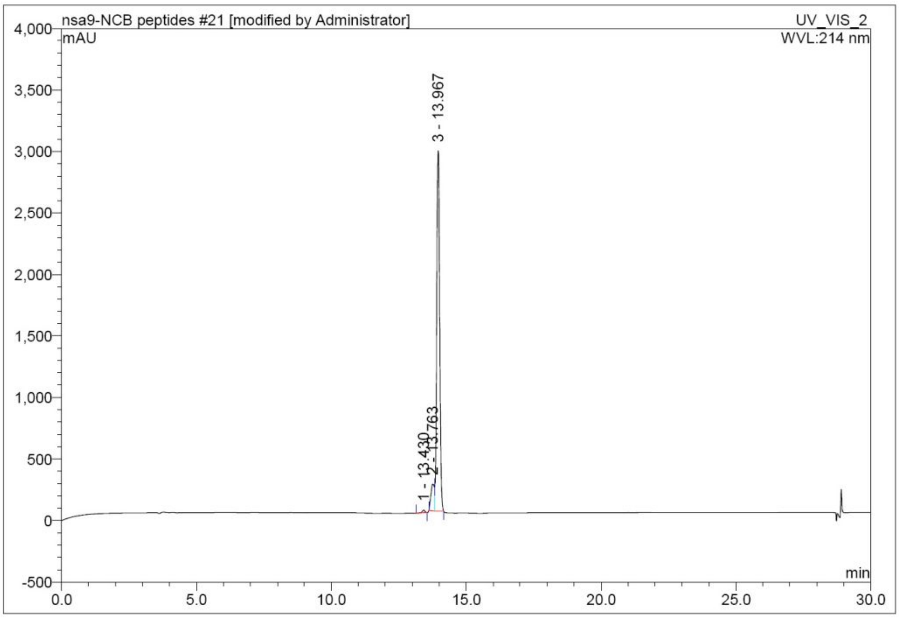

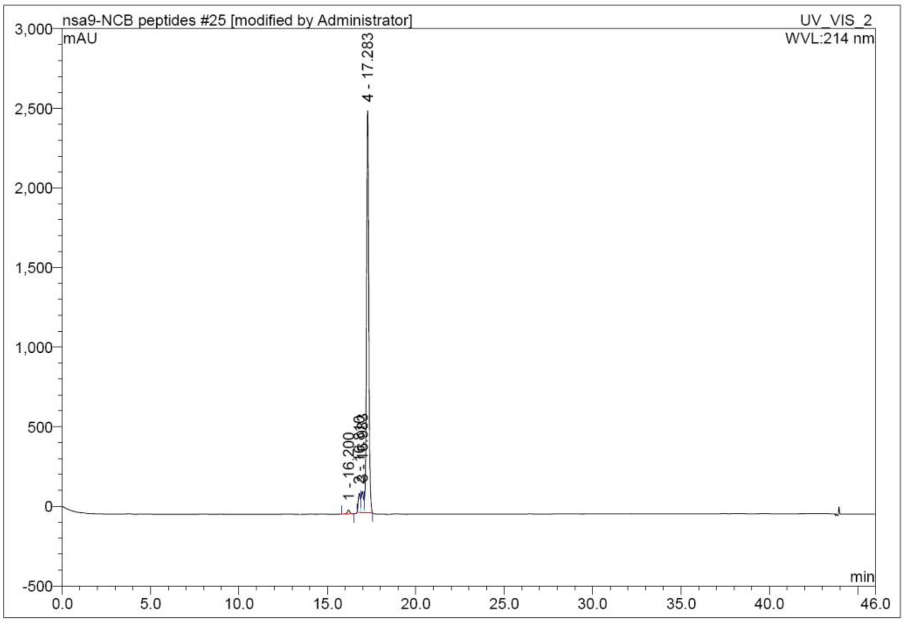

6 FTU-H4(4-10)K8Ac

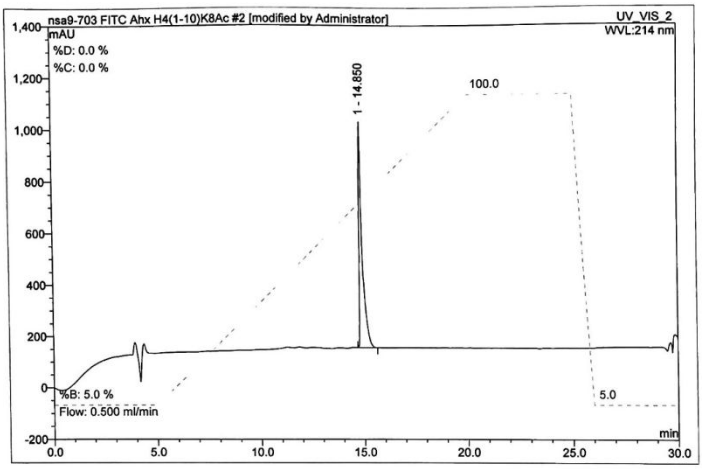

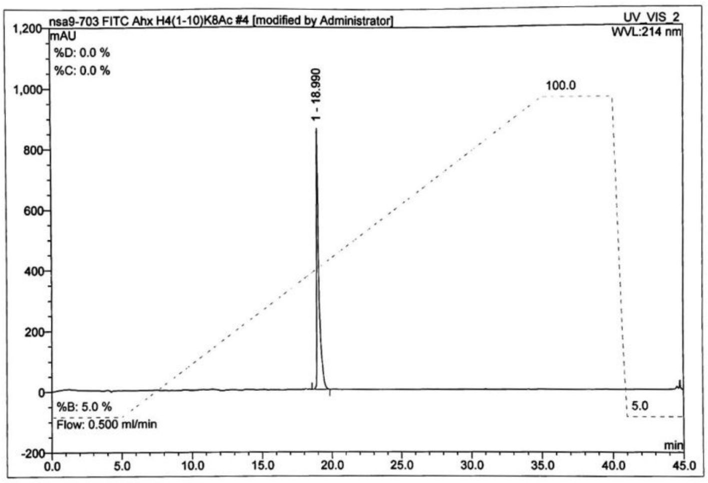

7 FTU-H4(9-15)K12Ac

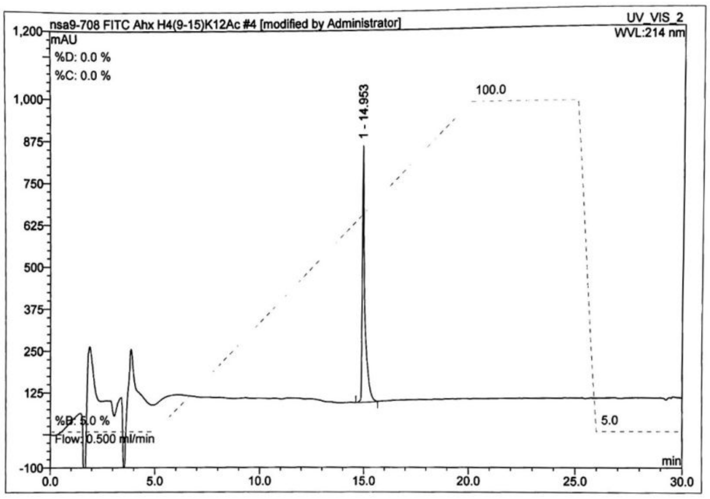

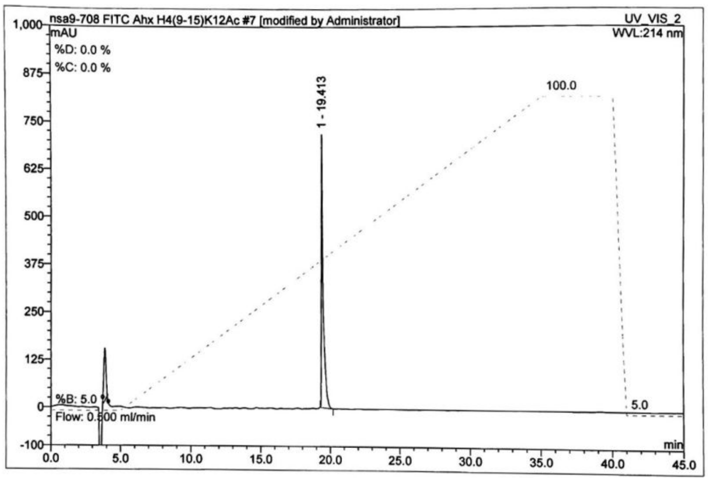

8 FTU-H4(12-18)K16Ac

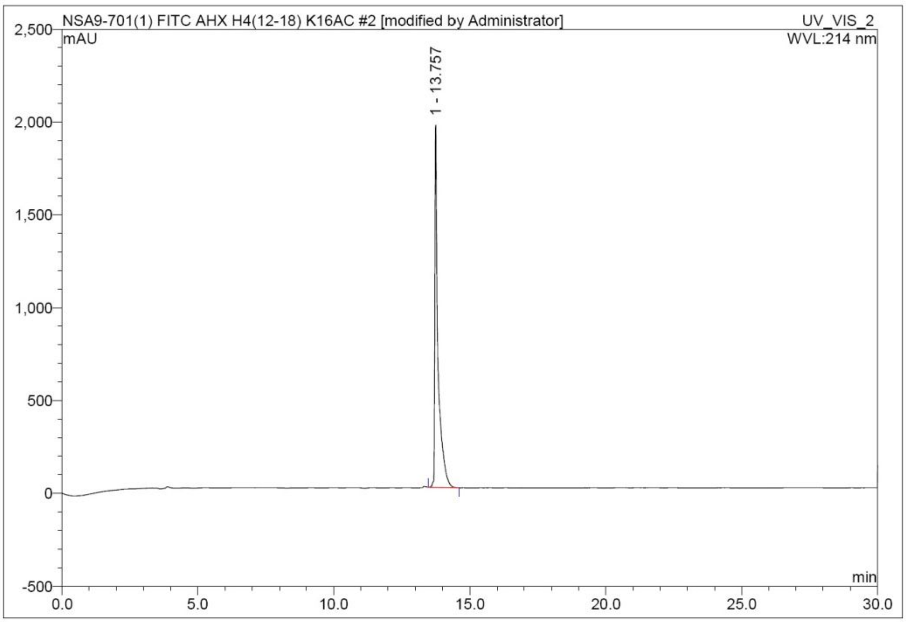

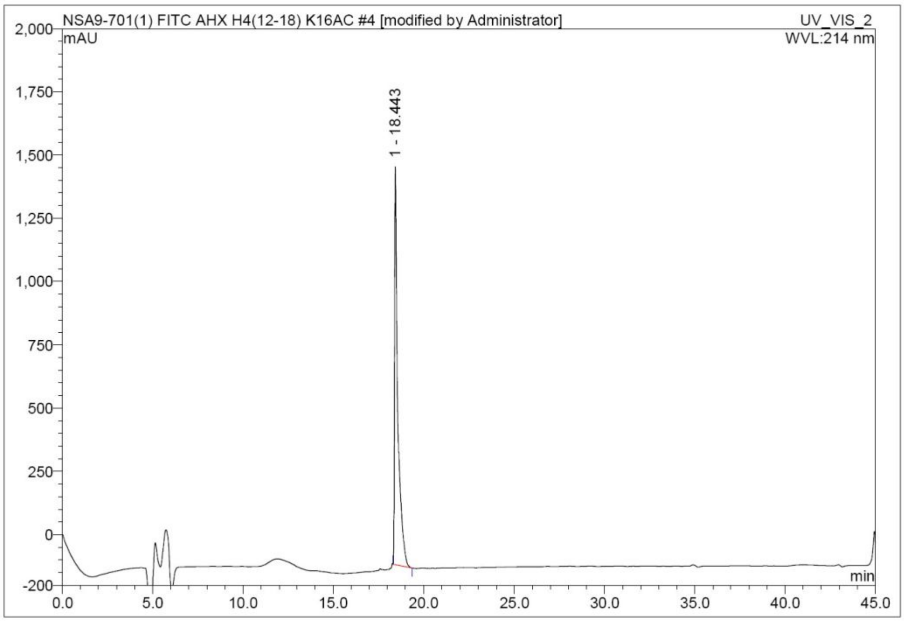

9 H3(1-7)K4Hd

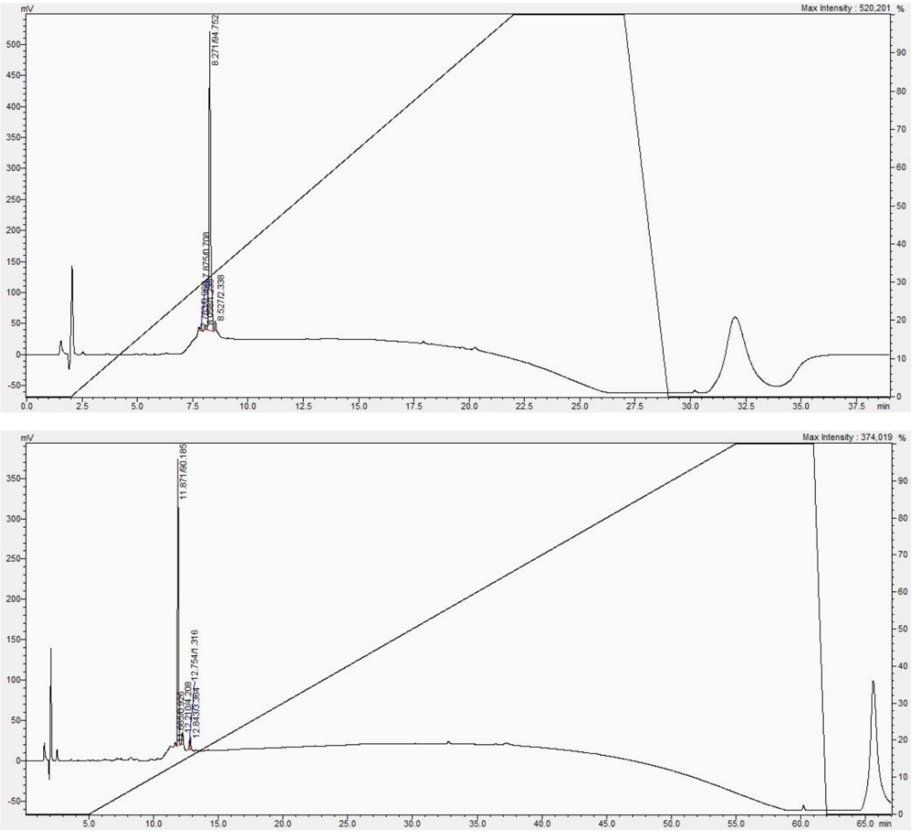
mV
Max Intensity : 520,201
%
8.271/94.752
7.875/0.708
8.527/2.338
min
Max Intensity : 374,019
11.871/90.185
12.754/1.316
min

10 H3(6-12)K9Hd

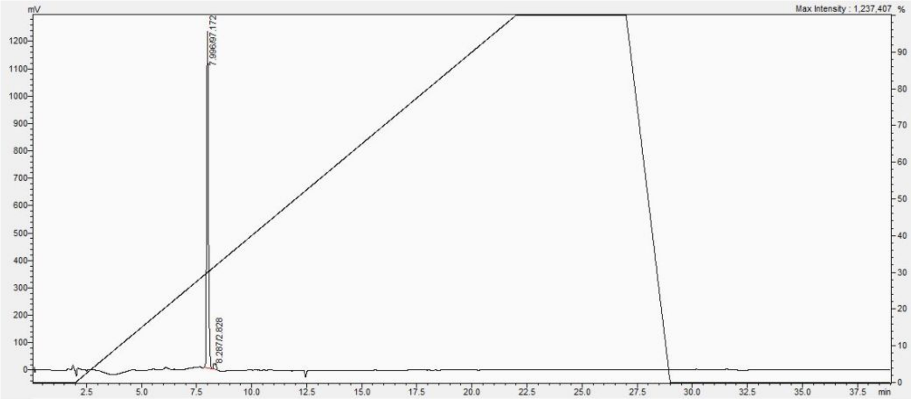

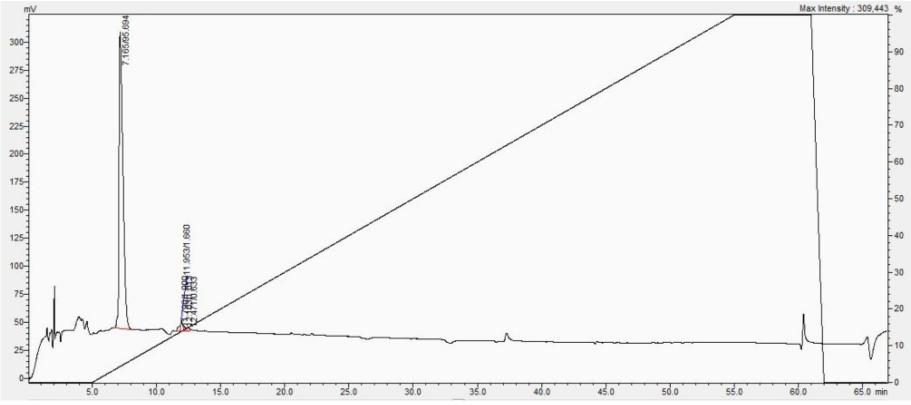

11 H3(11-17)K14Hd

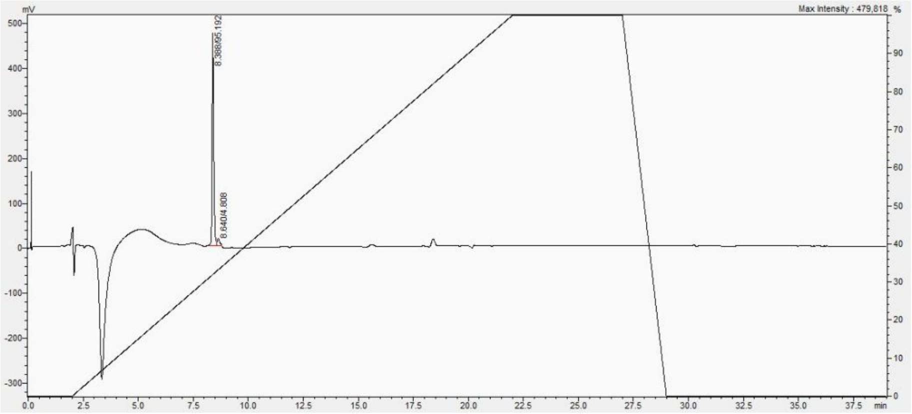

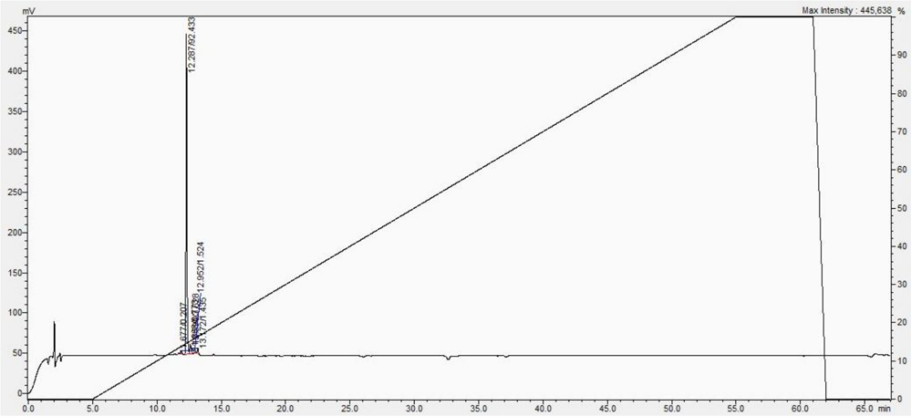

12 H3(23-29)K27Hd

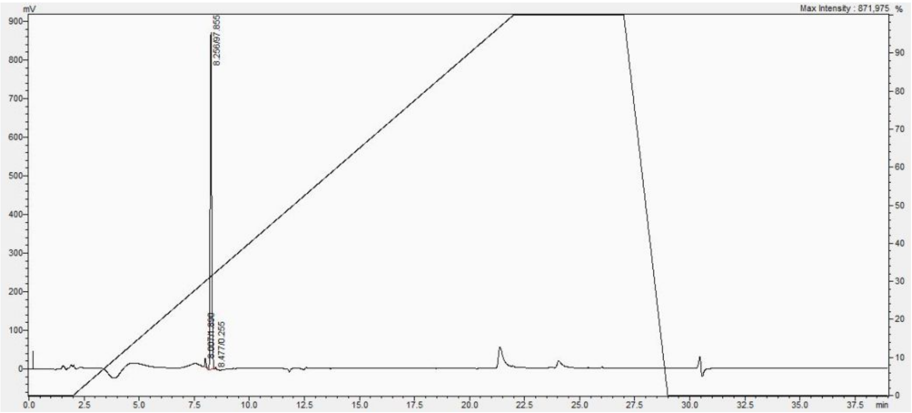

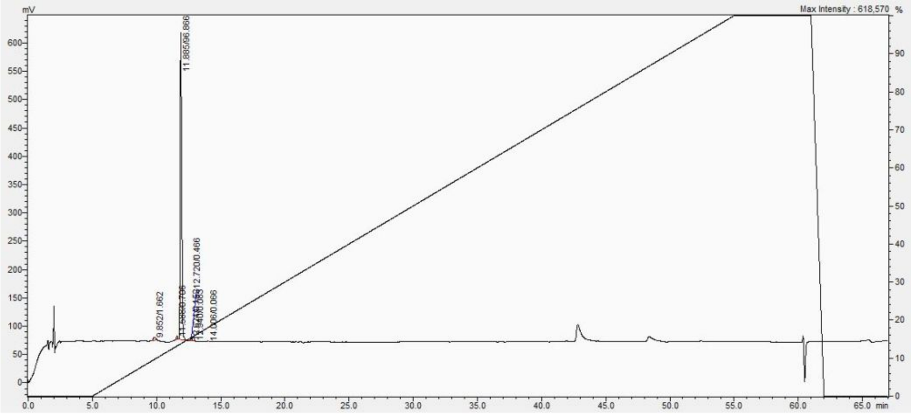

13 H4(1-8)K5Hd

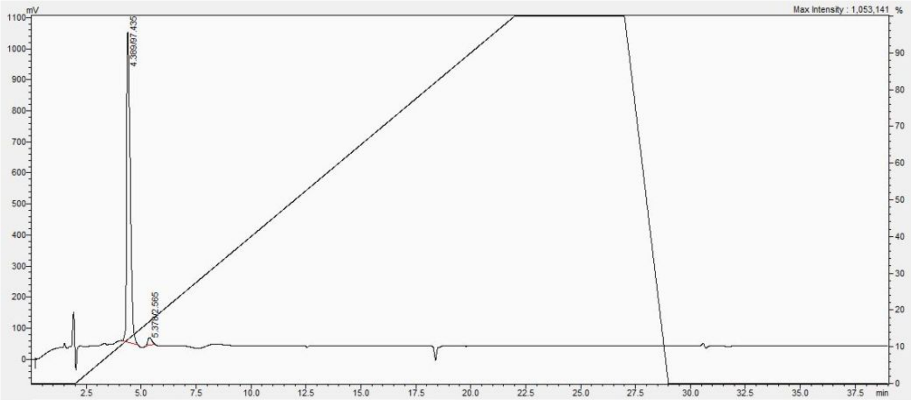

mV
Max Intensity : 1,053,141
%
4.389/97.435
5.376/2.565
min

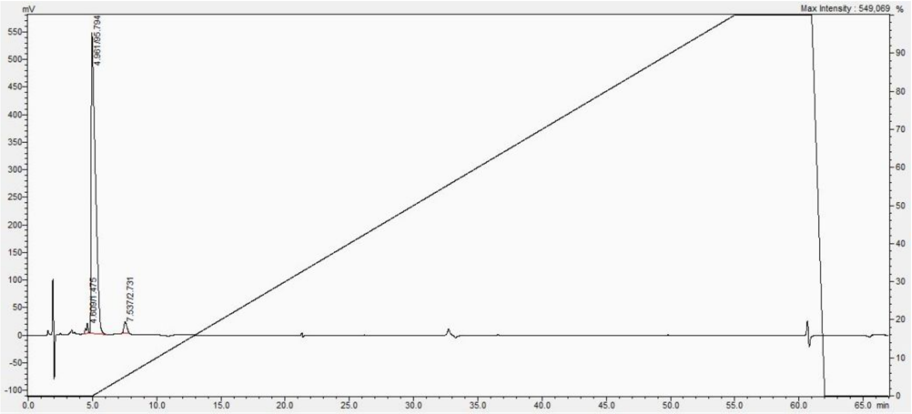

mV
Max Intensity : 549,069
%
4.961/95.794
4.609/1.475
7.537/2.731
min

14 H4(4-10)K8Hd

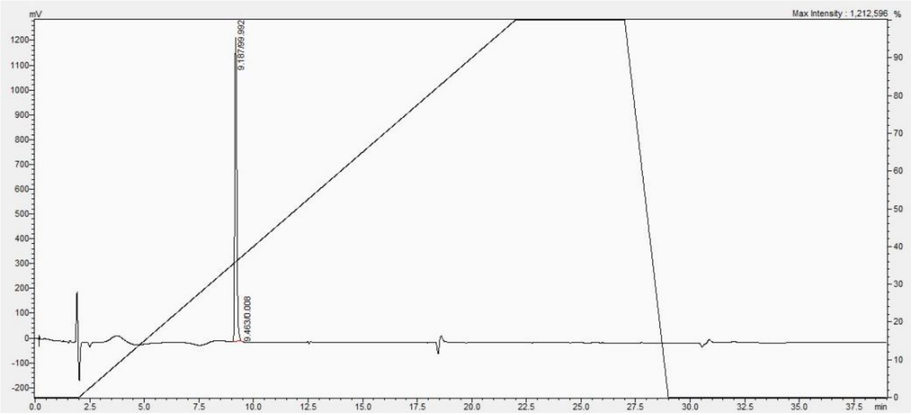

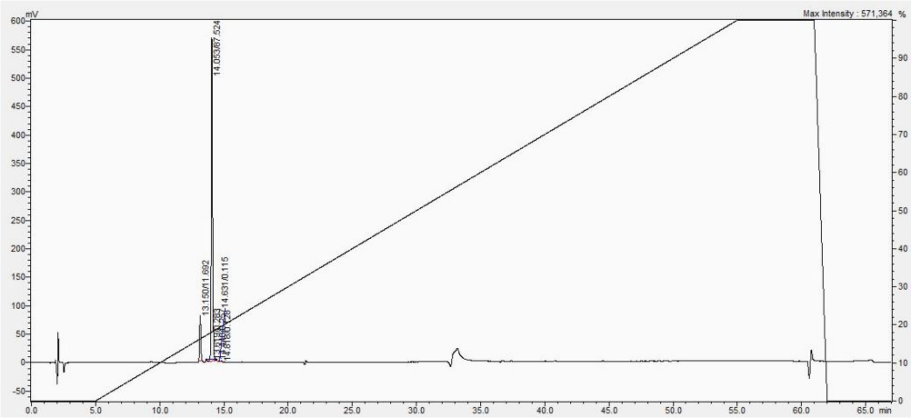

15 H4(9-15)K12Hd

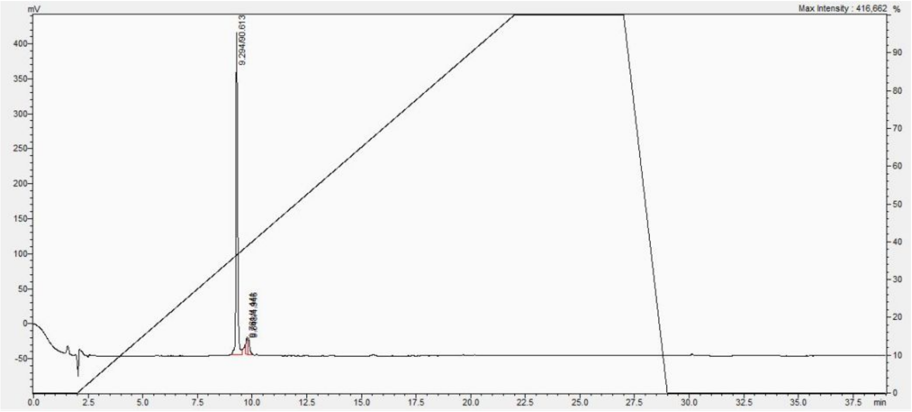

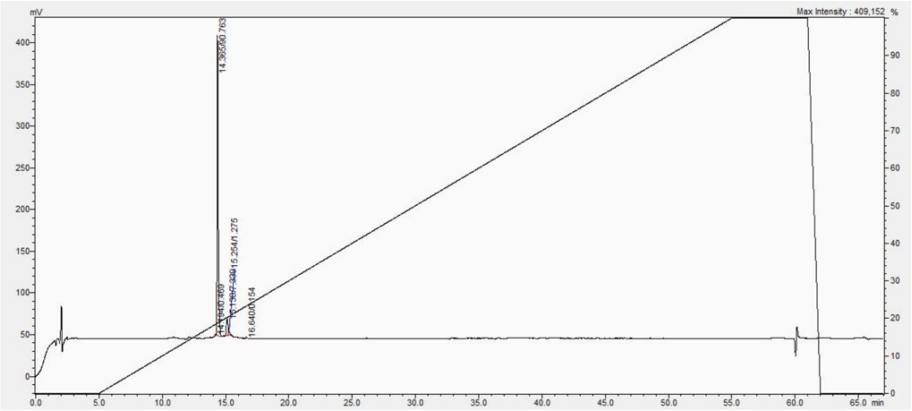

16 H4(12-18)K16Hd

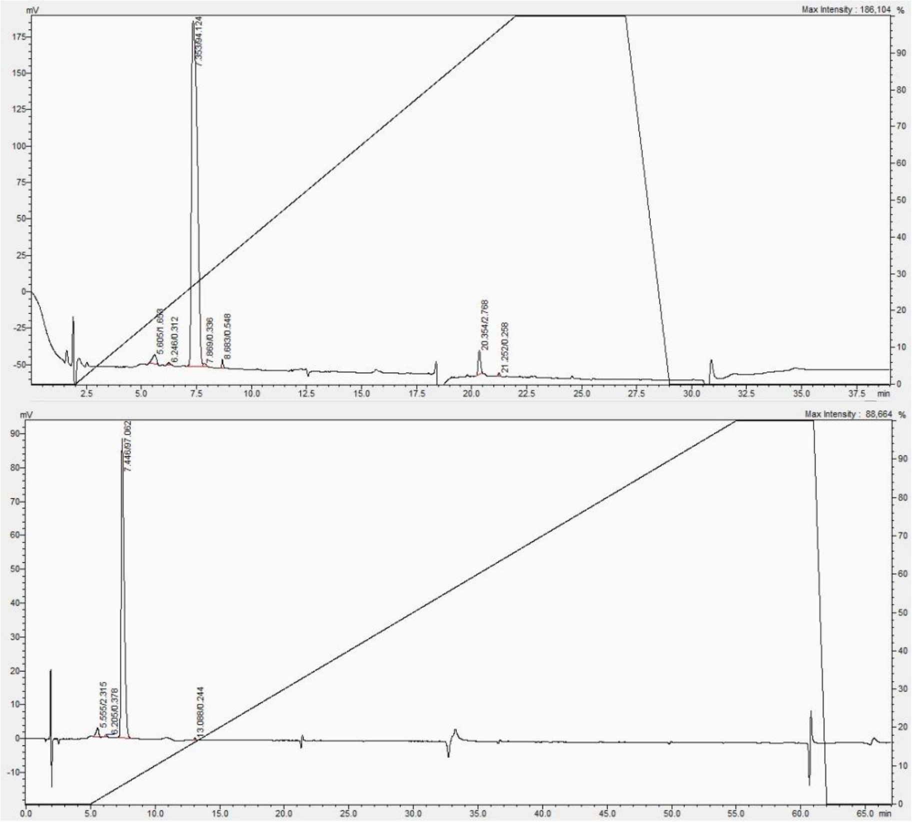

17 FTU-H3(5-11)K9Hd

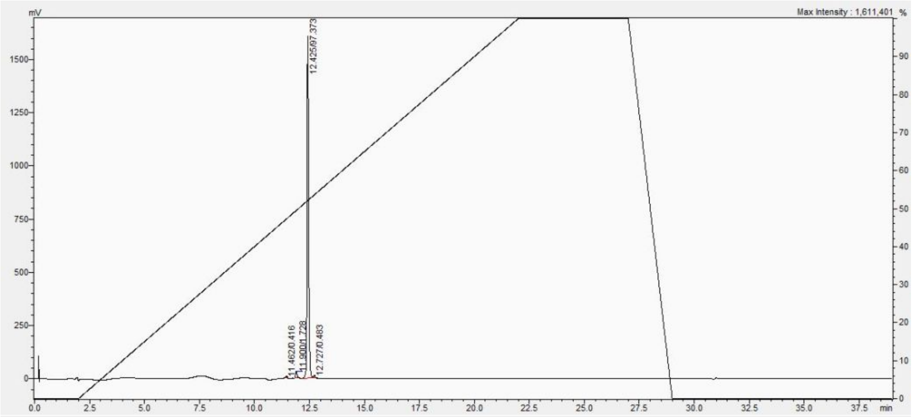

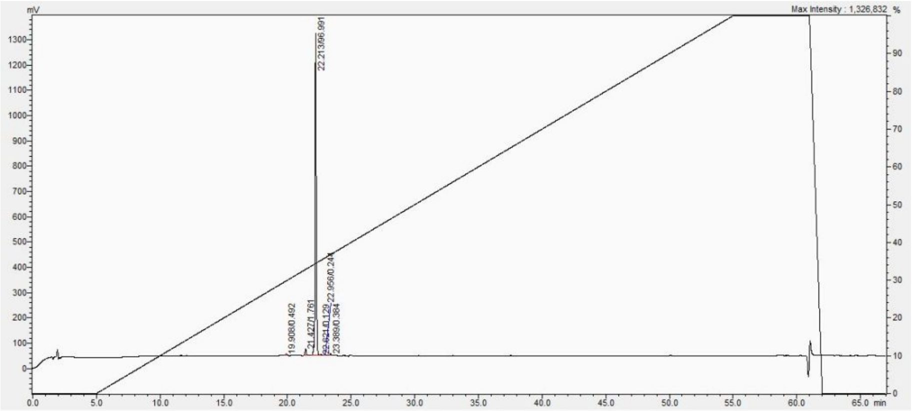

18 FTU-H3(23-29)K27Hd

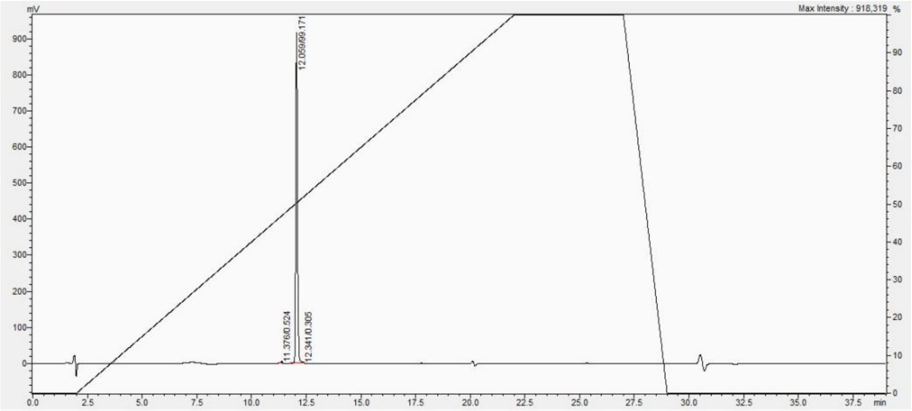

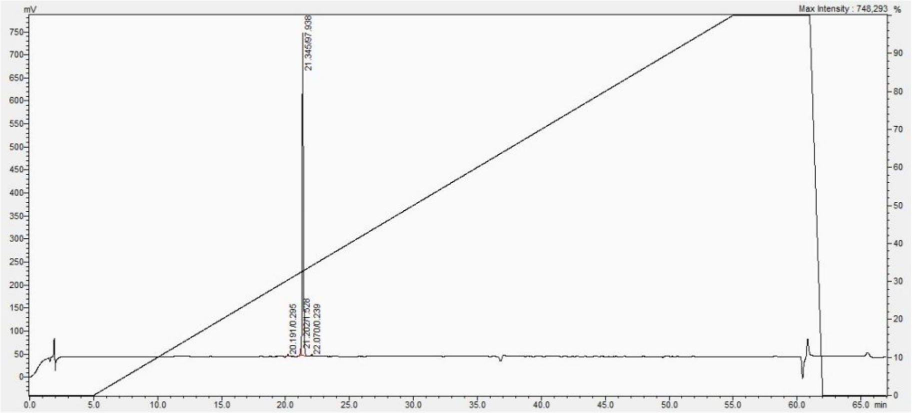

19 FTU-H3(8-14)K12Hd

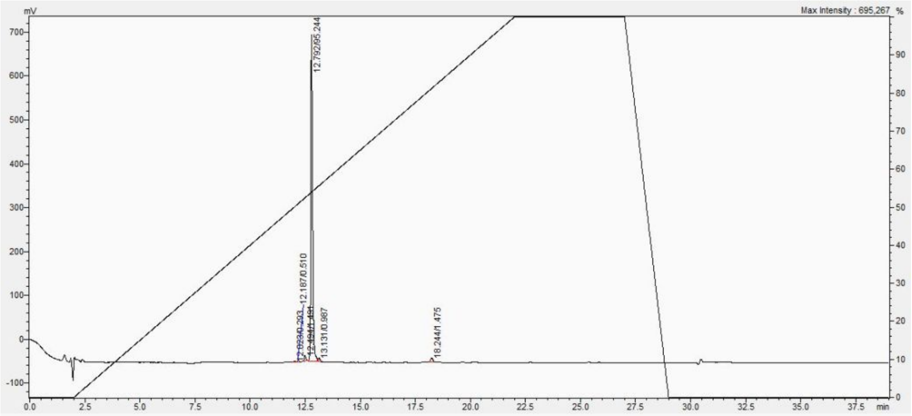

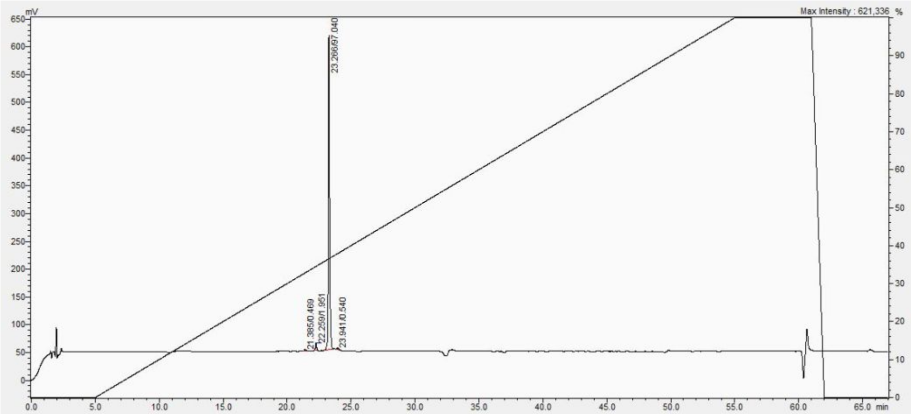

20 FTU-H4(12-18)K16Hd

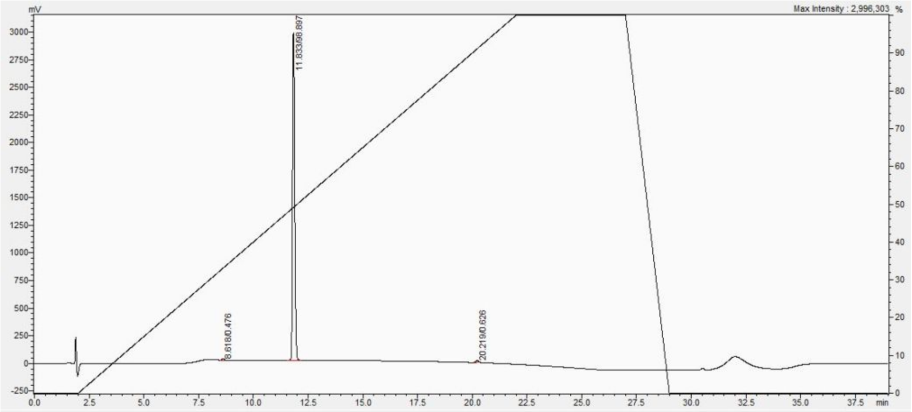

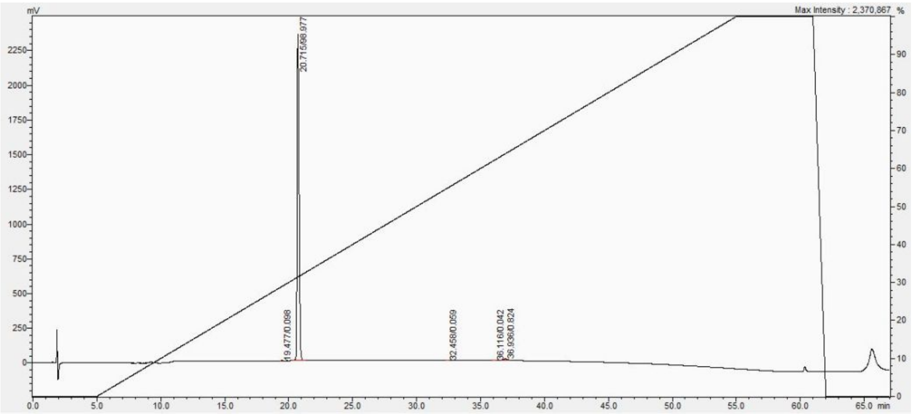

21 H4(8-14)K12Hd

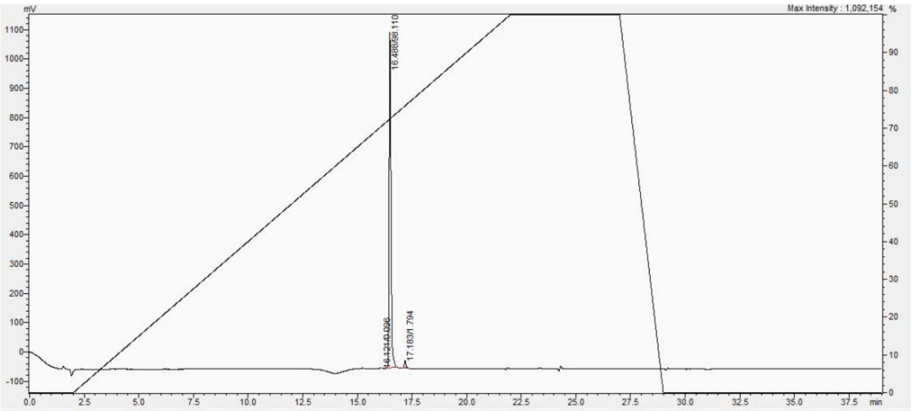

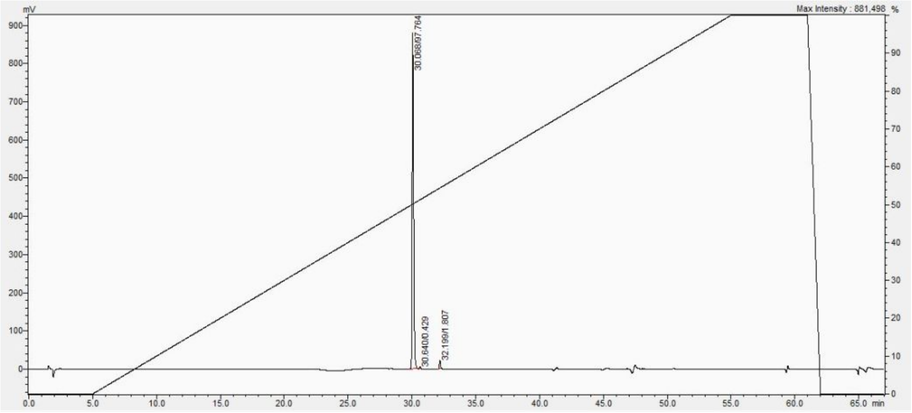

22 H4(8-14)L10GK12Hd

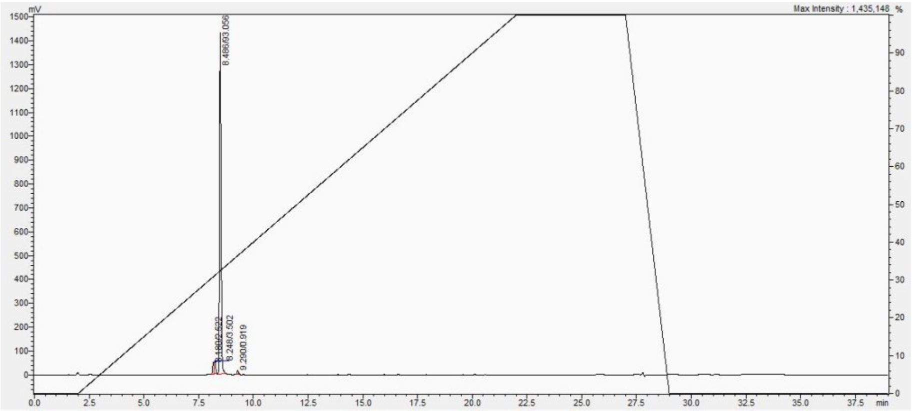

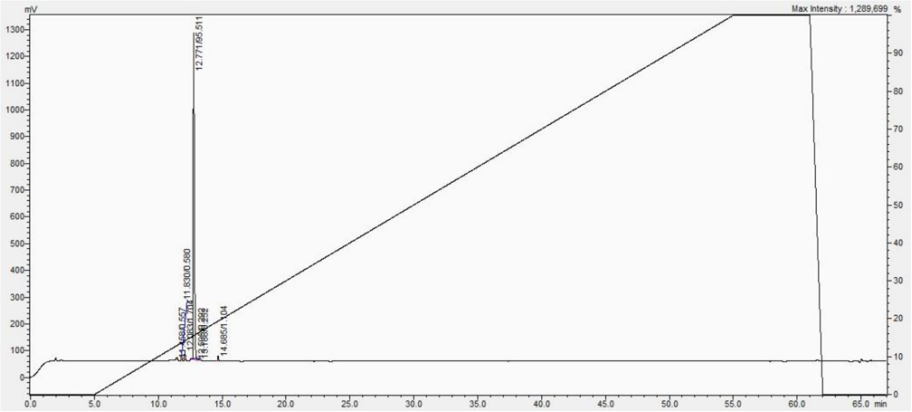

23 H3(23-29)K23AK27Hd

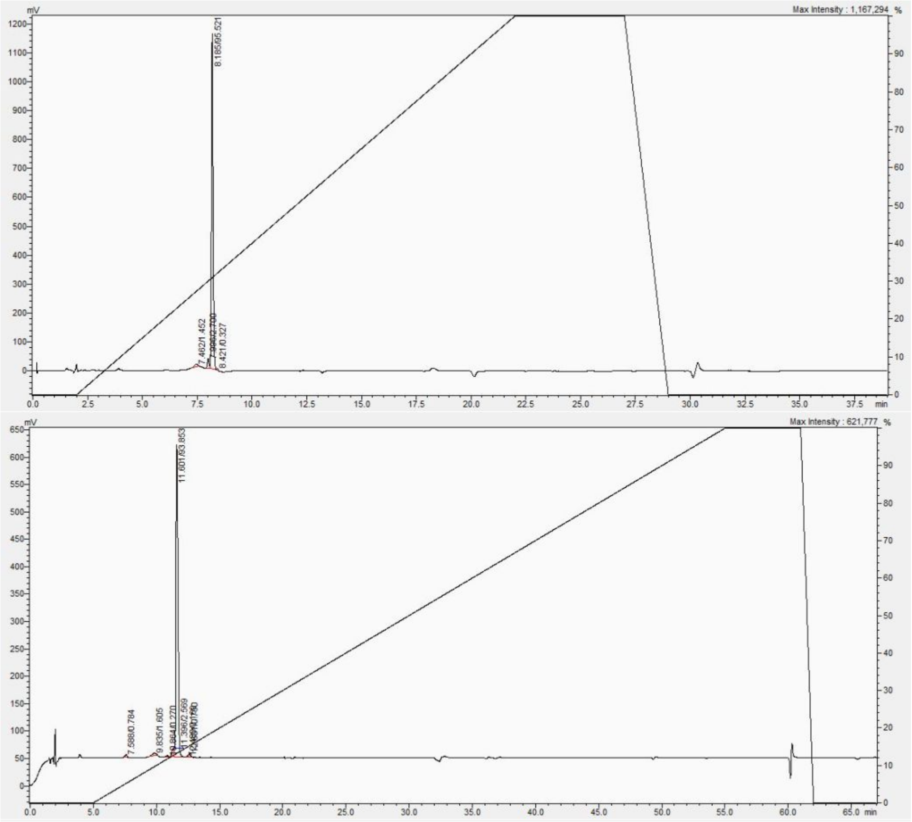

24 H3(23-29)R26AK27Hd

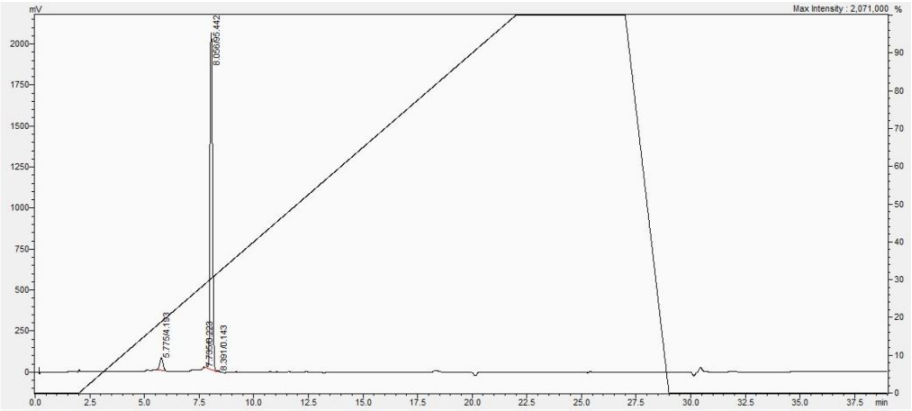

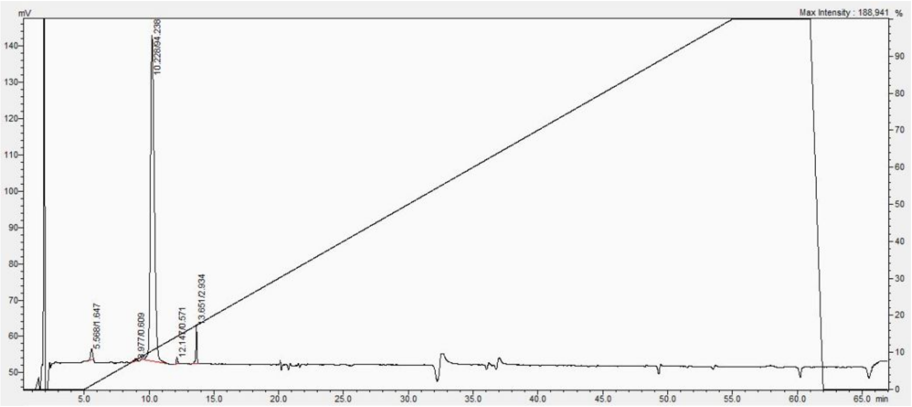

25 H3(23-29)S28AK27Hd

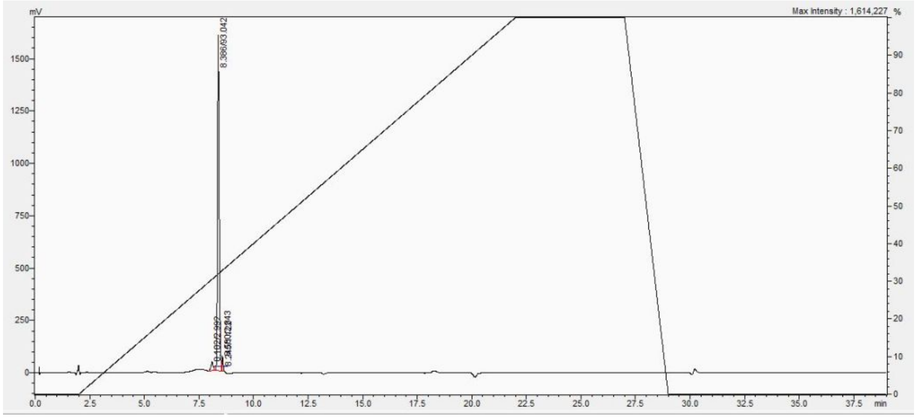

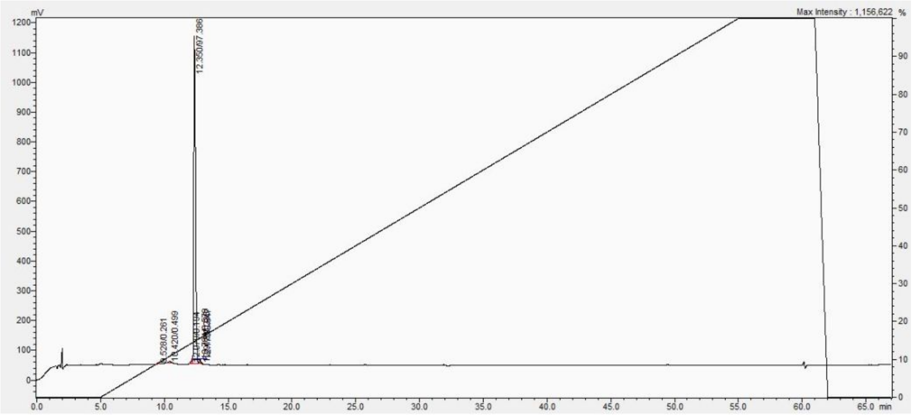

26 H4(12-18)K12AK16Hd

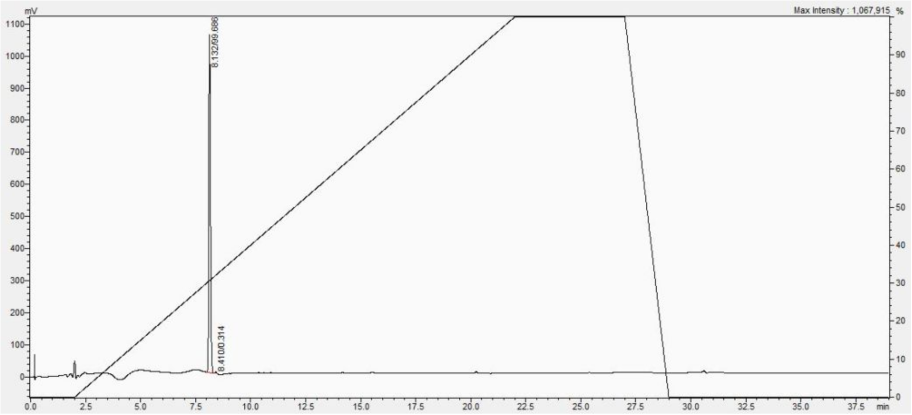

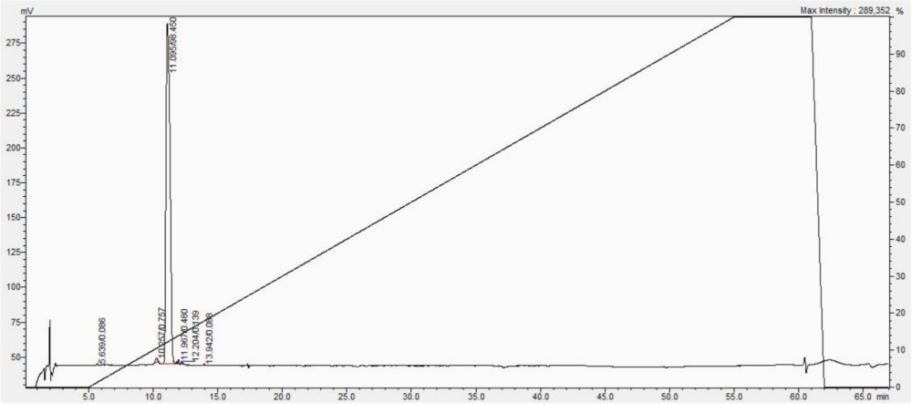

27 H4(12-18)R17AK16Hd

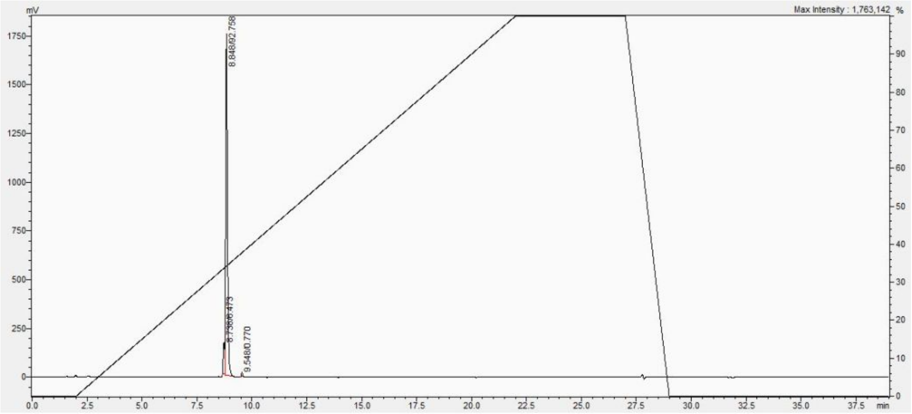

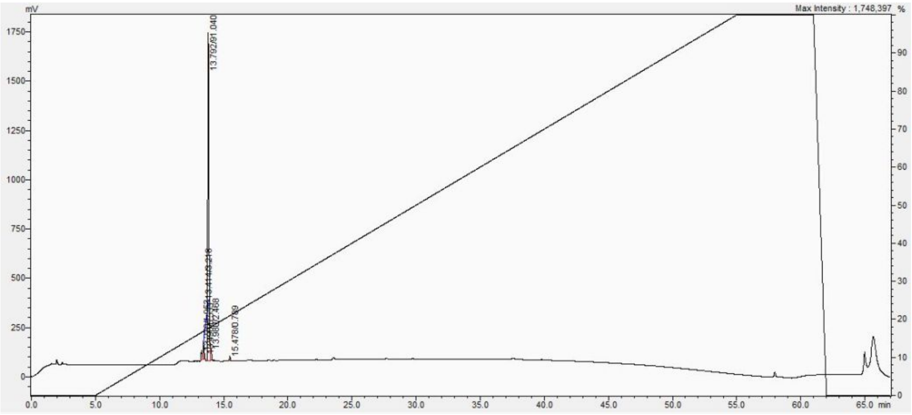

28 H4(12-18)H18AK16Hd

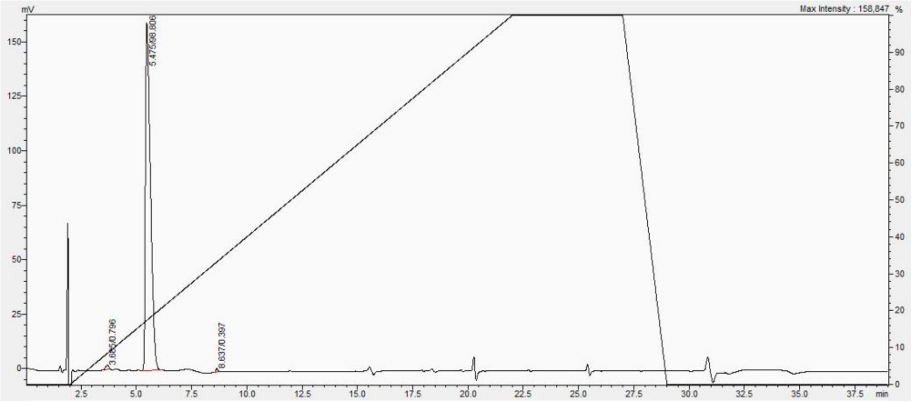

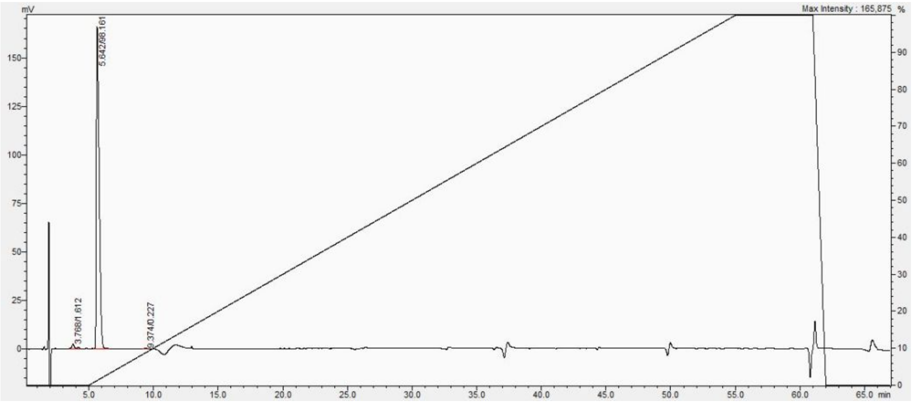

S.3 Table of Complex components

**HDAC Complexes**	**HDAC Co-regulator protein**	**HDAC**	**Additional Components**
RERE	RERE$^{(HAR)}$	HDAC1$^{(1-482)}$	
MIER1	MIER1$^{(1-512)}$	HDAC1$^{(1-482)}$	
CoREST	RCOR1$^{(86-485)}$	HDAC1$^{(1-482)}$	LSD1$^{(1-852)}$
NuRD	MTA1$^{(162-546)}$	HDAC1$^{(1-482)}$	RBBP4$^{(1-425)}$
MiDAC	MIDEAS$^{(628-887)}$	HDAC1$^{(1-482)}$	DNTTIP1$^{(1-329)}$
SIN3A	SIN3A$^{(461-1273)}$	HDAC1$^{(1-482)}$	RBBP4$^{(1-425)}$, SAP30L$^{(1-183)}$
SMRT	SMRT$^{(220-480)}$/GPS2$^{(1-49)(Chimera)}$	HDAC3$^{(1-428)}$	TBL1$^{(1-577)(F26A/I30A)(Dimer)}$

S.4 Table of Complex components

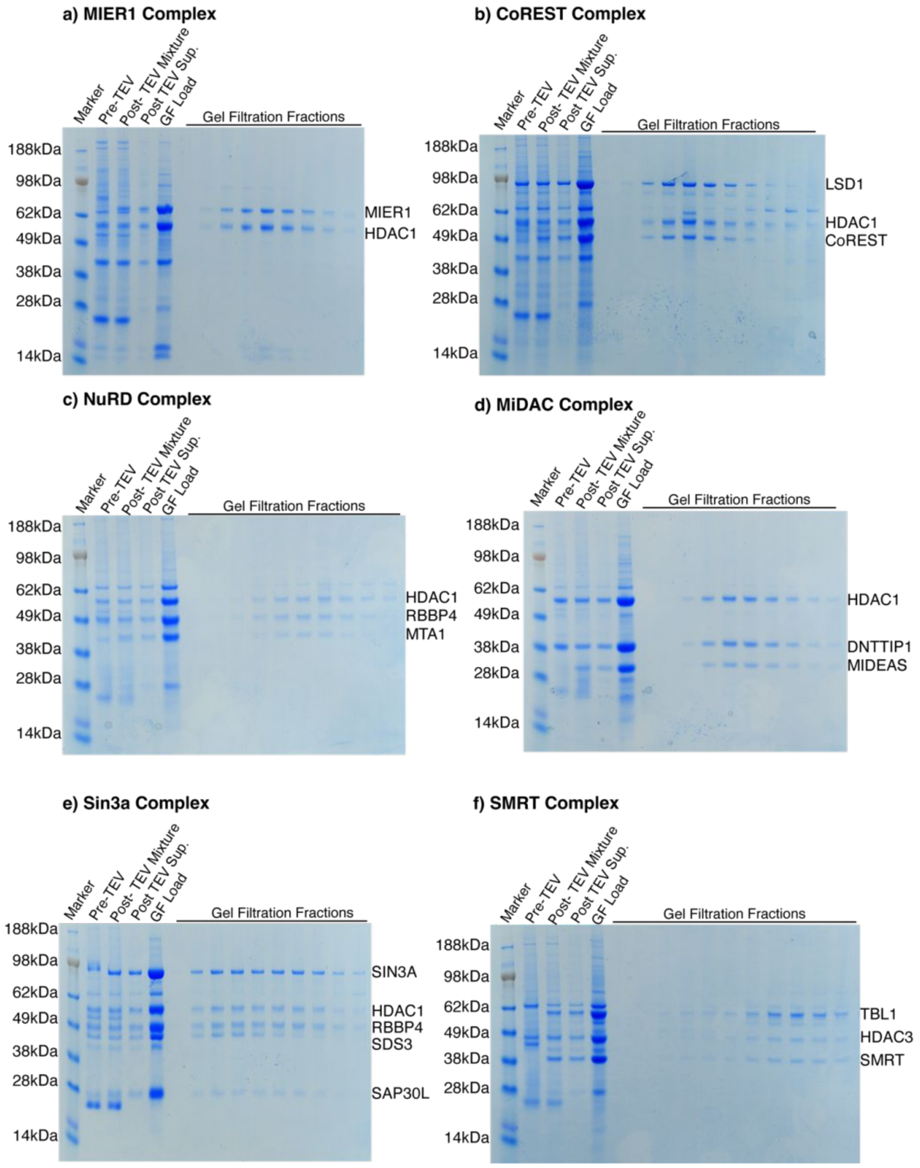

g) RERE Complex

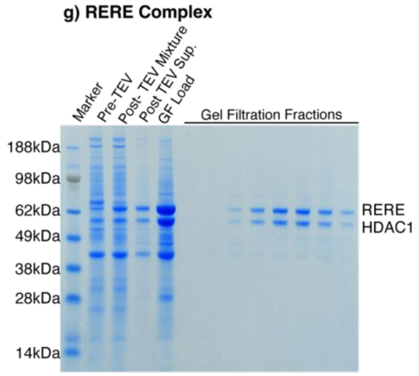

S.5 The turnover of acetyl peptides by HMR

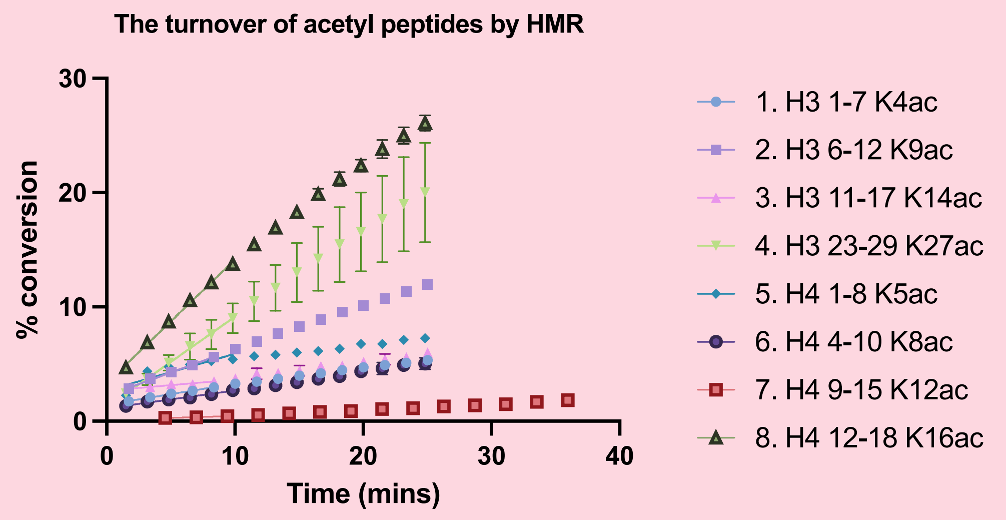

S.6 HMR against the peptide inhibitor library

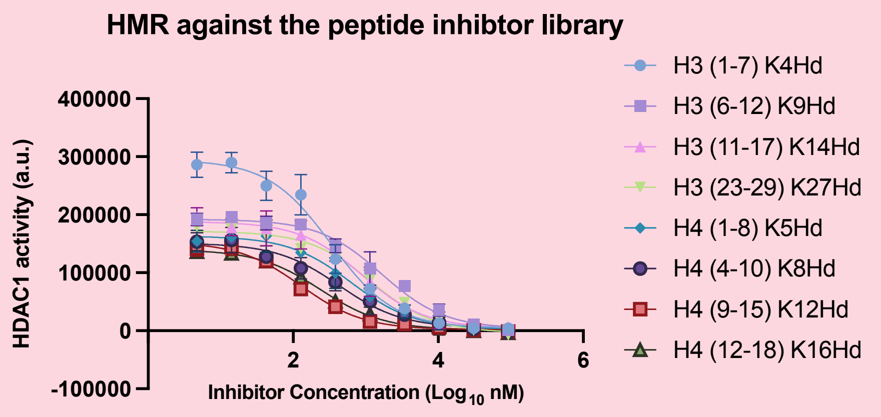

S.7 HMR against H3K27Hd Alanine Scan

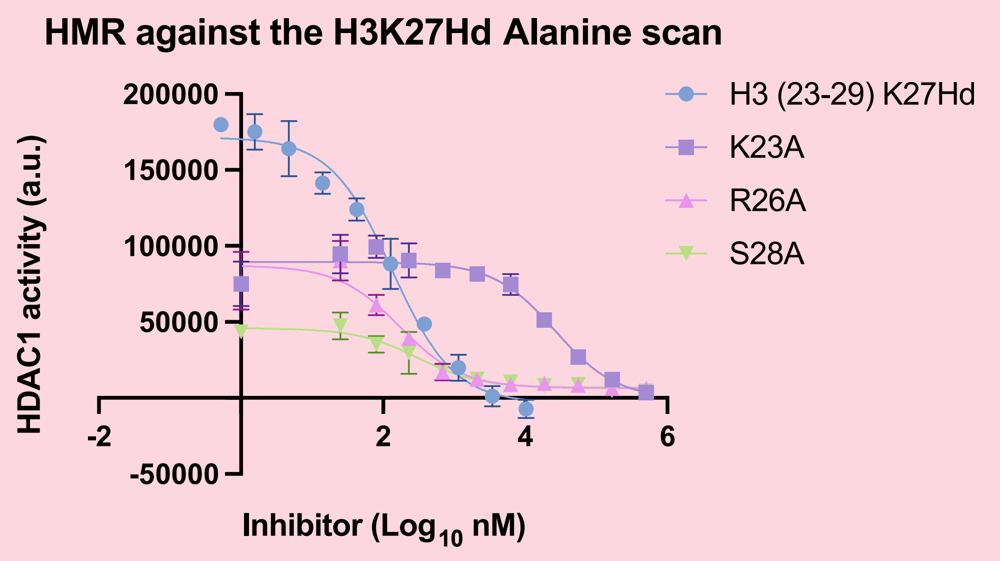

S.7 HMR against H4K16Hd Alanine Scan

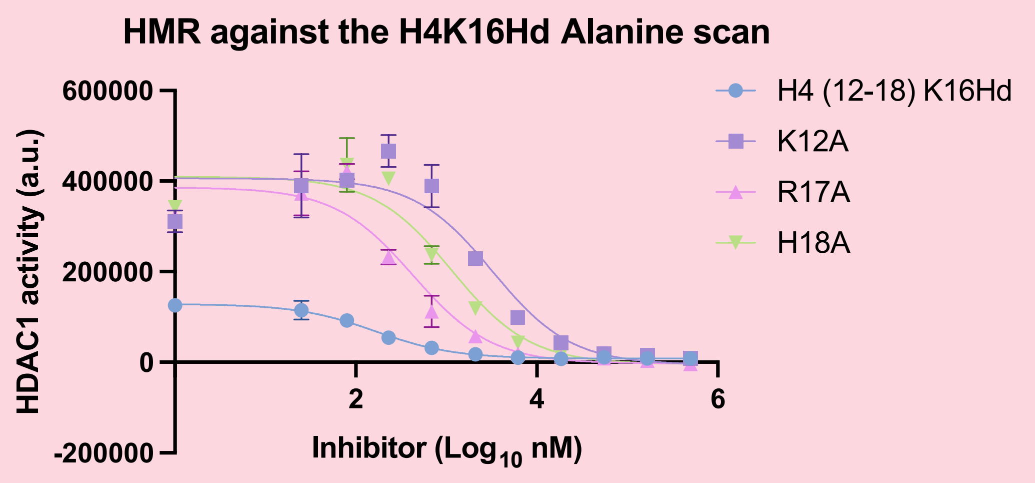

S.8 HMR against H4 K12Hd Peptides

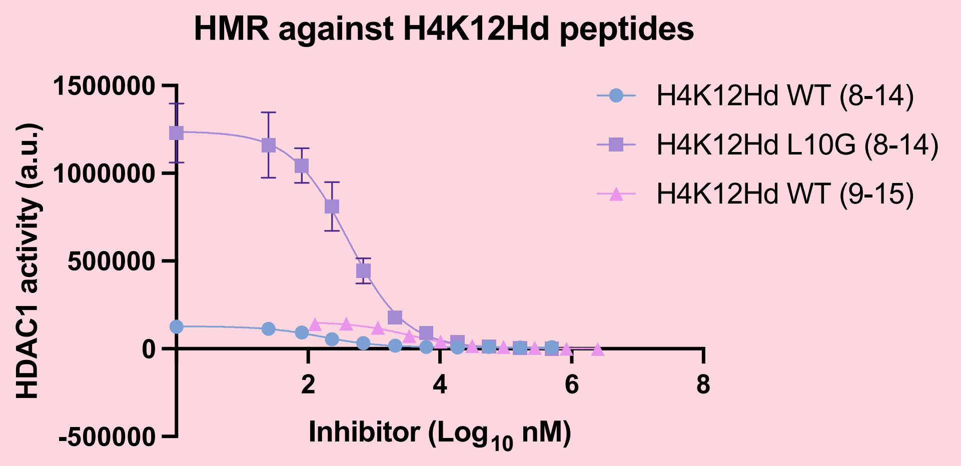

S.9 HDAC complexes against H3 (23-29) K27Hd

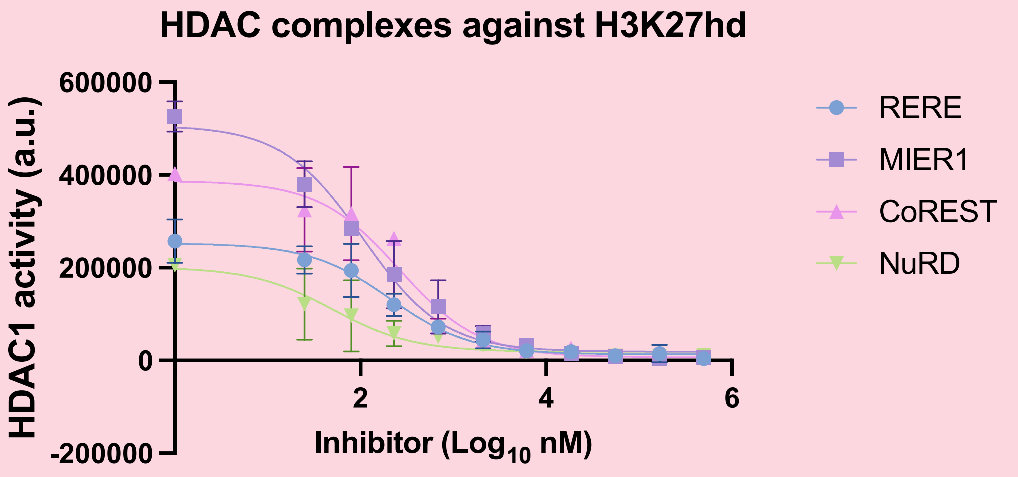

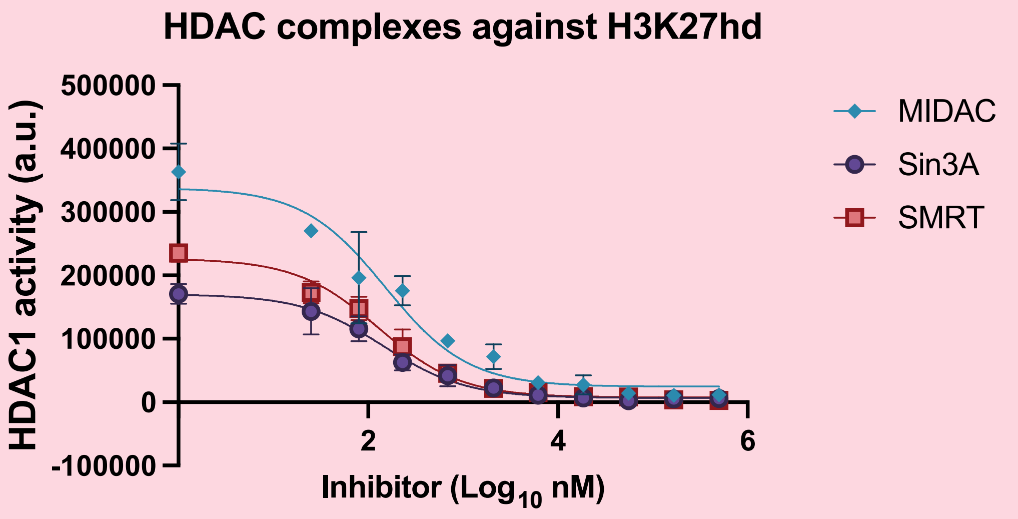

S.10 HDAC complexes against H4 (12-18) K16Hd

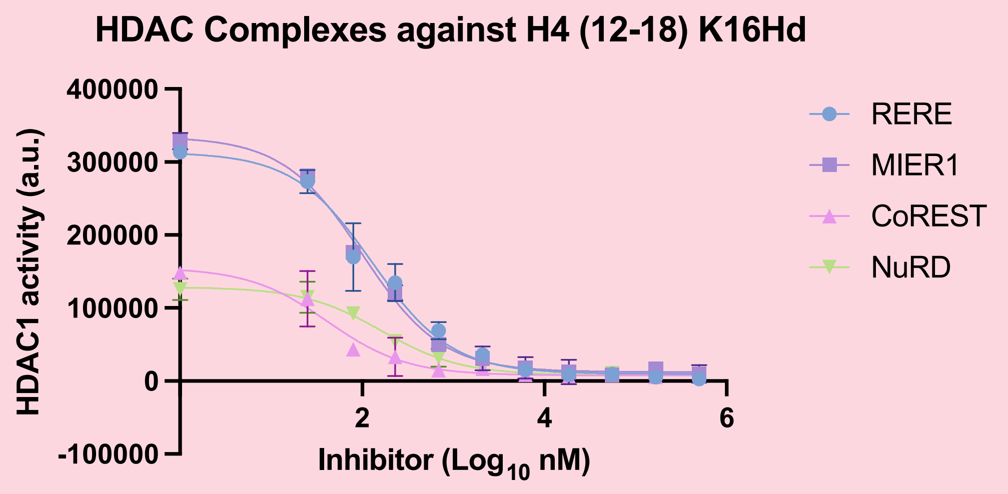

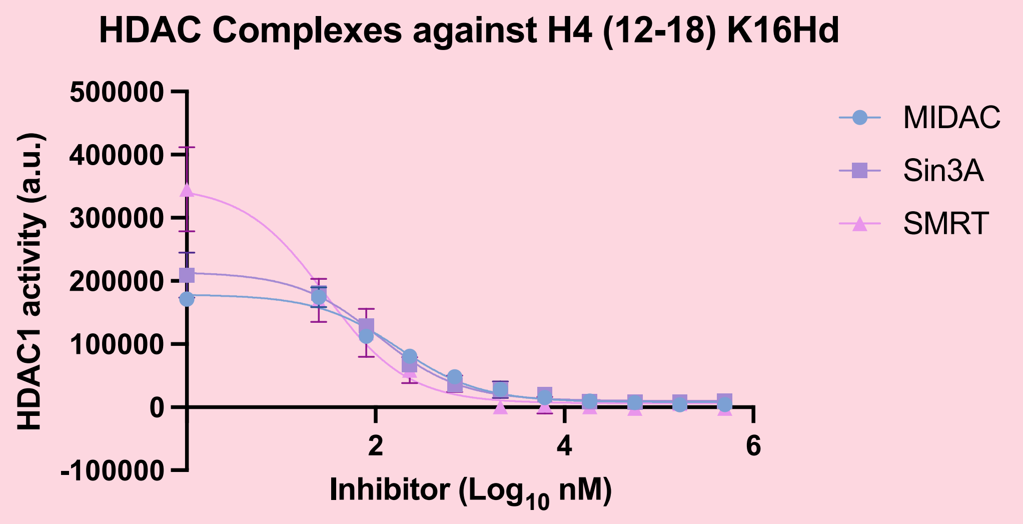

S.11 HDAC complexes against H3 (23-29) K27Hd K23A

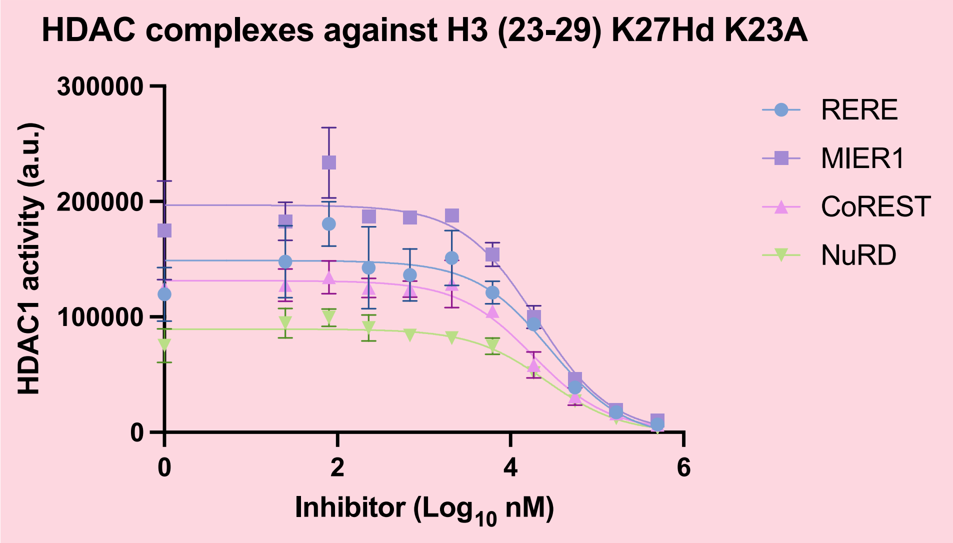

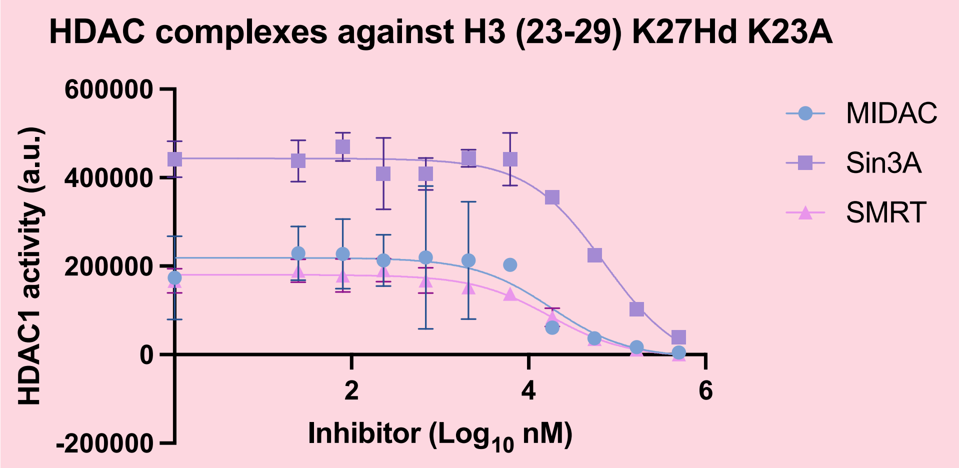

S.12 HDAC complexes against H3 (23-29) K27Hd R26A

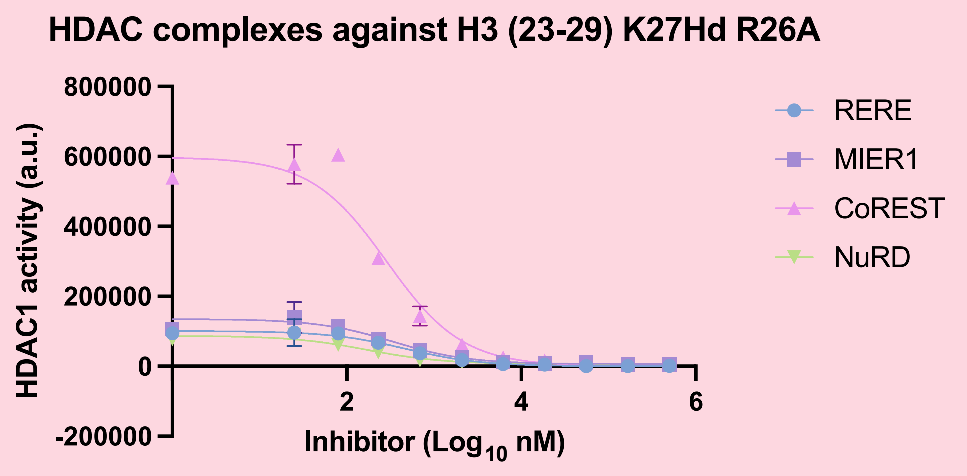

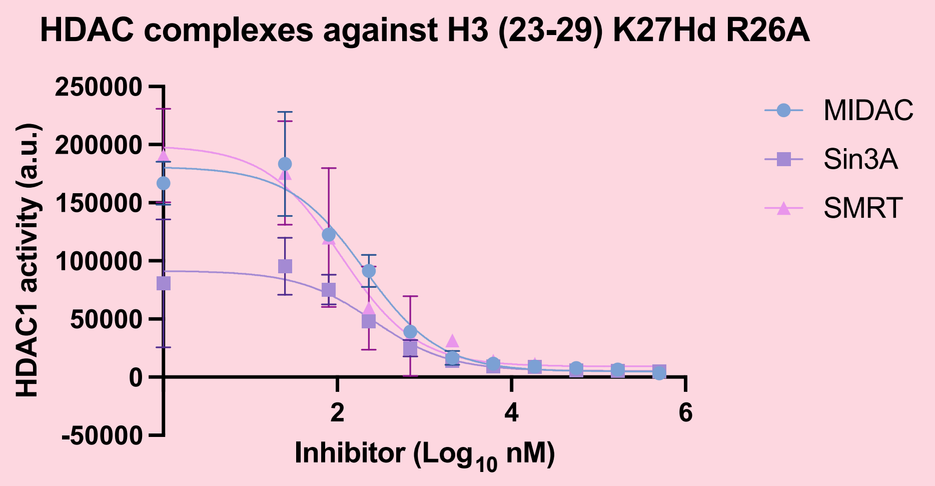

S.13 HDAC complexes against H3 (23-29) K27Hd S28A

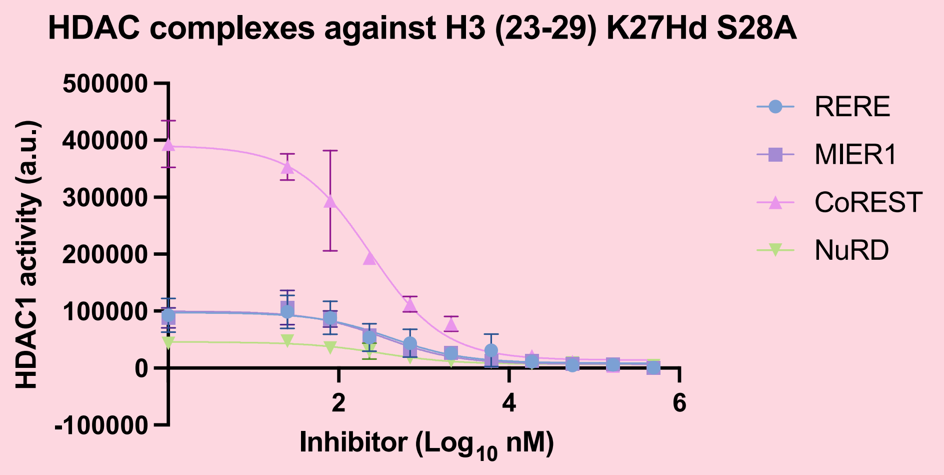

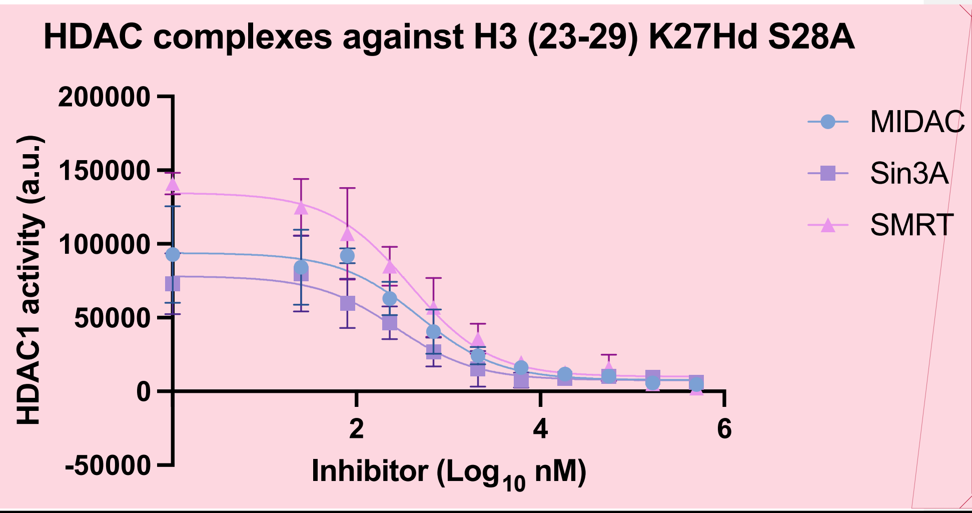

Commented [BEA1]: Inserted the Raw data graphs for all the experiments which are not shown in the main manuscript. This includes the:

% conversion v Time (Caliper Assay)

HDAC activity assay:
-HMR V peptide inhibitor library

-HMR Against H3K27Hd Alanine Scan

-HMR against H4K16Hd alanine scan

- HMR against H4K12Hd peptides

-HDAC complexes against H3K27Hd and H4K16Hd

- HDAC complexes against H3K27Hd Alanine Scan peptides.

S.14 Tables showing all of the initial rates and IC_{50}.

Initial Rate of HMR against histone peptides:

**Peptide**	**Rate (% conversion min^{-1})**	**Vmax**	**Km**
H3 1-7 K4ac	0.18 +/- 0.009	7.141	10.67
H3 6-12 K9ac	0.40 +/- 0.013	22.13	23.50
H3 11-17 K14ac	0.10 +/- 0.027	6.280	4.828
H3 23-39 K27ac	0.79 +/-0.075	81.99	77.88
H4 1-8 K5ac	0.32 +/-0.065	7.672	3.328
H4 4-10 K8ac	0.16 +/-0.016	9.120	22.16
H4 9-15 K12ac	0.03 +/-0.020	1.850e+014	Unstable
H4 12-18 K16ac	1.08 +/-0.024	52.66	26.54

The IC_{50} of HMR against histone peptide library

**Peptide**	**IC50 (nM)**
H3 1-7 K4Hd	333
H3 6-12 K9Hd	160
H3 11-17 K14Hd	886
H3 23-39 K27Hd	134
H4 1-8 K5Hd	633
H4 4-10 K8Hd	431
H4 9-15 K12Hd	3464
H4 12-18 K16Hd	240

The K_D of Fluorescein-labelled peptides against HMR

**Peptide**	**K_D (nM)**
H3 (6-12) K9Hd	98
H3 (23-29) K27Hd	60
H4 (8-14) K12Hd	288
H4 (12-18) K16Hd	84

The IC_{50} of HMR against H3 K27Hd, H4 K16Hd and H4K12Hd peptides

**Peptide**	**IC50 (nM)**
H3 (23-29) K27Hd	133
H3 (23-29) K27Hd K23A	26189
H3 (23-29) K27Hd R26A	172
H3 (23-29) K27Hd S28A	308
H4 (12-18) K16Hd	164
H4 (12-18) K16Hd K12A	3251
H4 (12-18) K16Hd R17A	429
H4 (12-18) K16Hd H18A	1150
H4 (9-15) K12Hd	3464
H4 (8-14) K12Hd	164
H4 (8-14) K12Hd L10G	403

HDAC Complexes against H3 K27Hd and H4 K16Hd

HDAC Complex	Peptide	IC50 (nM)
RERE	H3 (23-29) K27Hd	209
	H4 (12-18) K16Hd	130
MIER1	H3 (23-29) K27Hd	106
	H4 (12-18) K16Hd	100
CoREST	H3 (23-29) K27Hd	260
	H4 (12-18) K16Hd	41
HMR(NuRD)	H3 (23-29) K27Hd	50
	H4 (12-18) K16Hd	164
MiDAC	H3 (23-29) K27Hd	151
	H4 (12-18) K16Hd	176
Sin3a	H3 (23-29) K27Hd	145
	H4 (12-18) K16Hd	107
	H3 (23-29) K27Hd	128
SMRT	H4 (12-18) K16Hd	29

HDAC Complexes against H3K27Hd Alanine Scan peptides

HDAC Complex	Peptide	IC50 (nM)
RERE	H3 (23-29) K27Hd K23A	28712
	H3 (23-29) K27Hd R26A	464
	H3 (23-29) K27Hd S28A	408
MIER1	H3 (23-29) K27Hd K23A	20096
	H3 (23-29) K27Hd R26A	334
	H3 (23-29) K27Hd S28A	342
CoREST	H3 (23-29) K27Hd K23A	18284
	H3 (23-29) K27Hd R26A	287
	H3 (23-29) K27Hd S28A	234
HMR (NuRD)	H3 (23-29) K27Hd K23A	26189
	H3 (23-29) K27Hd R26A	172
	H3 (23-29) K27Hd S28A	308
MiDAC	H3 (23-29) K27Hd K23A	18090
	H3 (23-29) K27Hd R26A	203
	H3 (23-29) K27Hd S28A	480
Sin3a	H3 (23-29) K27Hd K23A	71650
	H3 (23-29) K27Hd R26A	260
	H3 (23-29) K27Hd S28A	272
SMRT	H3 (23-29) K27Hd K23A	17425
	H3 (23-29) K27Hd R26A	110
	H3 (23-29) K27Hd S28A	379

Commented [BEA2]: Add a table showing all of the initial rates, Kd and IC50 for all of the experiments
